# Microbiological and Molecular Assessment of Bacteriophage ISP for the Control of *Staphylococcus aureus*

**DOI:** 10.1371/journal.pone.0024418

**Published:** 2011-09-09

**Authors:** Katrien Vandersteegen, Wesley Mattheus, Pieter-Jan Ceyssens, Florence Bilocq, Daniel De Vos, Jean-Paul Pirnay, Jean-Paul Noben, Maia Merabishvili, Urszula Lipinska, Katleen Hermans, Rob Lavigne

**Affiliations:** 1 Division of Gene Technology, Katholieke Universiteit Leuven, Heverlee, Belgium; 2 Department of Pathology, Bacteriology and Poultry Diseases, Ghent University, Merelbeke, Belgium; 3 Hasselt University, Biomedical Research Institute and Transnational University Limburg, School of Life Sciences, Diepenbeek, Belgium; 4 Laboratory for Molecular and Cellular Technology, Burn Centre, Queen Astrid Military Hospital, Brussels, Belgium; 5 Eliava Institute of Bacteriophage, Microbiology and Virology, Tbilisi, Georgia; University of Edinburgh, United Kingdom

## Abstract

The increasing antibiotic resistance in bacterial populations requires alternatives for classical treatment of infectious diseases and therefore drives the renewed interest in phage therapy. Methicillin resistant *Staphylococcus aureus* (MRSA) is a major problem in health care settings and live-stock breeding across the world. This research aims at a thorough microbiological, genomic, and proteomic characterization of *S. aureus* phage ISP, required for therapeutic applications. Host range screening of a large batch of *S. aureus* isolates and subsequent fingerprint and DNA microarray analysis of the isolates revealed a substantial activity of ISP against 86% of the isolates, including relevant MRSA strains. From a phage therapy perspective, the infection parameters and the frequency of bacterial mutations conferring ISP resistance were determined. Further, ISP was proven to be stable in relevant *in vivo* conditions and subcutaneous as well as nasal and oral ISP administration to rabbits appeared to cause no adverse effects. ISP encodes 215 gene products on its 138,339 bp genome, 22 of which were confirmed as structural proteins using tandem electrospray ionization-mass spectrometry (ESI-MS/MS), and shares strong sequence homology with the ‘Twort-like viruses’. No toxic or virulence-associated proteins were observed. The microbiological and molecular characterization of ISP supports its application in a phage cocktail for therapeutic purposes.

## Introduction

The scientific reappraisal of the use of bacteriophages in the treatment of bacterial infections is reflected by hundreds of phage therapy-related publications in the last decade. However, so far, no phage preparation has been approved for market authorization. In 2009, Merabishvili *et al.* [1] evaluated the safety and efficacy of bacteriophage therapy with the standardized quality-controlled small-scale production of the phage cocktail BFC-1. This cocktail contains two phages, 14/1 and PNM, infecting *Pseudomonas aeruginosa*, and one phage infecting *Staphylococcus aureus*, both frequent pathogens in burn wound infections. The *S. aureus*-infecting phage in BFC-1 is ISP, a member of the *Myoviridae* and closely related to phage G1 [2]. ISP was originally isolated in the 1920s from an unknown source in Tbilisi (Georgia) by the Eliava Institute of Bacteriophage, Microbiology and Virology and was selected as a therapeutic phage based on a host range study on burn wound isolates. The physicochemical properties and the pyrogenicity of the phage cocktail, and hence of the ISP preparation, are conform to the European Pharmacopoeia standards and show no cytotoxicity towards human neonatal foreskin keratinocytes. The quality control of BFC-1 also confirmed the absence of temperate bacteriophages and verified the presence of the expected virion morphology as well as the specific interaction with the target bacteria [1].

In this paper, we present the complete microbiological and molecular examination of this therapeutically important phage, which includes stability assays, genome and virion analysis and an extensive host range screening.

## Analysis

### ISP host range screening and analysis of the *Staphylococcus* host collection

High-titer ISP stocks were obtained through amplification in liquid Mueller Hinton medium using *S. aureus* subsp. *aureus* Rosenbach ATCC 6538 (further referred to as ‘strain ATCC 6538’). Subsequent purification and concentration of the phage was performed by CsCl density gradient centrifugation following polyethylene glycol 8000 precipitation [3].

**Figure 1:**
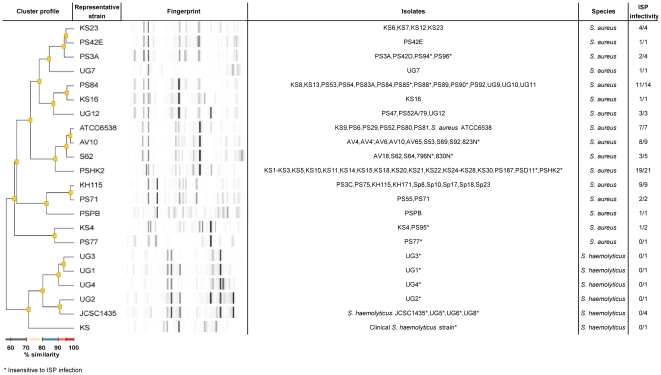
Dendrogram of the 22 identified *S. aureus* and *S. haemolyticus* genotypes. The dendrogram was generated based on rep-PCR patterns using a 98% similarity level. For each genotype, the representative strain and its fingerprint, the constituting isolates and the ISP sensitivity are indicated.

Phage ISP was subjected to a host screening involving 86 *S. aureus* strains and nine *S. haemolyticus* isolates (Table S1). These isolates have a different origin, ranging from human and animal isolates to propagation strains for typing phages. All isolates were typed using automated repetitive sequence-based PCR (rep-PCR) DNA fingerprinting. Therefore, bacterial DNA was isolated with the UltraClean™ Microbial DNA Isolation Kit (MO Bio Laboratories, Carlsbad, USA) and rep-PCR was performed using the DiversiLab™ DNA *Staphylococcus* fingerprinting Kit (bioMérieux, Brussels, Belgium). In a next step, rep-PCR profiles were obtained using the microfluidic DNA chips (DiversiLab™ LabChip, bioMérieux) and an Agilent 2100 BioAnalyzer (Agilent Technologies, USA) according to the manufacturer's instructions. The resulting rep-PCR fingerprinting profiles were compared using the web-based DiversiLab software (bioMérieux), version 3.3. The *S. aureus* and *S. haemolyticus* isolates were clustered into 22 genotypes (98% similarity) (Figure 1). The *S. aureus* and the *S. haemolyticus* isolates are clearly clustered separately. Further, the human clinical *S. aureus* isolates (N = 34) and the phage propagation strains (N = 31) were quite randomly distributed over 14 clusters. The horse (N = 6), pig (N = 3) and poultry (N = 5) isolates grouped into two closely related clusters and the rabbit (N = 7) isolates clustered together with two phage propagation strains.

For a subset of human and animal isolates, the presence of genes encoding resistance to commonly used antimicrobial agents was determined with DNA array hybridization using the StaphyType96 Kit (Alere Technologies GmbH, Jena, Germany) (Table S1). All tested isolates carry tetracycline resistance genes and all but one isolate encode methicillin resistance and penicillinase genes. Further, genes encoding resistance to MLS (macrolide-lincosamide-streptogramin) antimicrobials and aminoglycosides are present in 14 and 19 isolates, respectively. These resistance profiles illustrate the clinical relevance of the target strains. DNA array hybridization also revealed the presence of bacterial toxin genes, for example toxic shock toxin, leukocidins and enterotoxins. The subset of analyzed isolates includes members of Epidemic MRSA (EMRSA) clones Rhine-Hesse EMRSA/UK-EMRSA-3 (KS6, KS7), North German/Iberian EMRSA (UG9, UG10, UG11, UG12), Lyon Clone/UK-EMRSA-2 (KS8, KS13) and Berlin EMRSA (KS1, KS2, KS3, KS4, KS5, KS10, KS11, KS14). These EMRSA clones are widespread and represent hospital-acquired, carrying ‘staphylococcal chromosomal cassette’ (SCC) *mec* element types I, II and III, as well as community-acquired, carrying SCC*mec* types IV and V, MRSA [4], [5]. In addition, all pig, poultry and horse isolates are members of the emerging MRSA clonal complex CC398, the most prevalent strain of livestock-associated MRSA [6], [7].

The host range was examined by spotting a tenfold serial dilution of an ISP stock on a Mueller Hinton soft agar lawn containing the potential host. The results were confirmed by a plaque assay [8], which permitted to assess the efficiency of plating, the relative phage titer on a bacterial strain compared to the maximum titer observed. ISP infects 86% of the *S. aureus* strains and none of the *S. haemolyticus* strains. All 34 human *S. aureus* isolates were infected by ISP, while nine phage propagation strains are insensitive to ISP infection. Interestingly, none of the pig strains, but all other animal strains are sensitive to ISP infection. Moreover, all but one of the *S. aureus* strains isolated from humans and rabbits were infected by ISP with a high efficiency of plating, whereas ISP displays a moderate efficiency of plating on six of the sensitive phage propagation strains. In addition, the efficiency of plating varies among the horse strains and is mainly low on the poultry strains.

No correlation was observed between the 22 genotypes and ISP sensitivity nor between the subset of characterized EMRSA clones and ISP sensitivity. This is consistent with previous observations made for fifteen *Pseudomonas aeruginosa* phages and their infection patterns on 94 AFLP-typed host strains [9]. Nonetheless, the lytic activity of ISP on all *S. aureus* strains isolated from patients is promising for phage therapy purposes.

### ISP infection parameters and emergence of bacterial resistance

**Figure 2:**
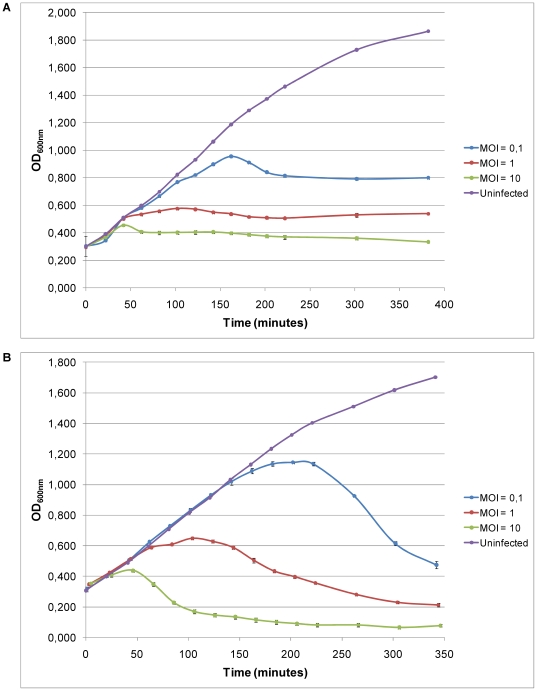
Infection curves of *Staphylococcus* phage ISP. Three independent experiments of a non-infected culture and cultures infected with MOI 0.1, 1 and 10 of *S. aureus* phage propagation strain 47 (A) and *S. aureus* subsp. *aureus* Rosenbach ATCC 6538 (B) are shown. Standard deviations are indicated.

In addition to infectivity on agar plates, the capability of phage ISP to adsorb on and subsequently infect *S. aureus* in a liquid culture at 37°C was examined. The optical density at 600 nm of two *S. aureus* strains, phage propagation strain 47 and strain ATCC 6538, infected at different multiplicities of infection (MOI, the ratio of phage particles to host cells) was monitored during a certain time period. Both infection curves suggest a lytic cycle of approximately 40 minutes. Interestingly, these killing curves progress differently in time, as shown in Figure 2. Phage ISP is able to completely lyse a culture of phage propagation strain 47 at MOI 10 and for two hours this bacterial culture remains stable. In contrast, *S. aureus* subsp. *aureus* Rosenbach ATCC 6538 is only attenuated by ISP. At MOI 0.1, 1 and 10 the optical density at 600 nm stabilizes at 0.8, 0.5 and 0.3, respectively. Monitoring of the number of free phages, adsorbed phages and viable bacteria during the experiment hints at a lysis inhibition phenomenon (data not shown). However, this long-delayed lysis mechanism for virion yield maximizing could not be substantiated, since no increased burst size was observed. For adsorption experiments, samples were taken at fixed intervals and transferred to Mueller Hinton medium supplemented with chloroform to lyse the remaining bacteria [8], [10]. Titration of the amount of nonadsorbed or reversibly adsorbed phages showed that ISP adsorption occurs quite rapidly. Approximately 50% of the ISP particles were bound within 1 minute and after 25 minutes a maximal adsorption of 85% was reached (Figure S1).

For five human isolates, the rate at which ISP-resistant *S. aureus* cells arise, was determined using the method of Beale [11] with small adjustments. Therefore, 300 µl of bacterial cultures grown in liquid Mueller Hinton medium was mixed with an excess of ISP particles and plated out. Following 48 hours of incubation at 37°C, resistant colonies were counted. As with the *P. aeruginosa* phage phiKMV [12], resistant colonies of various sizes were observed. Interestingly, the ISP mutation rate (Table S2) varies greatly between the different human isolates. *S. aureus*-resistant colonies were observed a hundred times less frequent than phage-resistant mutants of *Streptomyces erythreus* and *Streptococcus thermophilus* in a similar experiment [13], [14]. Additionally, ten randomly selected resistant colonies of each human isolate were purified two times by single-colony isolation and challenged again with ISP, to verify their resistance. 44 out of 50 (88%) colonies appeared to have acquired stable ISP resistance and hence the presented mutation rates are slightly overestimated. The appearance of phage-resistant mutants can be countered by using a complementary phage cocktail [15].

### Biophysical stability and *in vivo* safety of ISP

To assess the stability of phage ISP for storage and manipulation of the phage preparation as well as future animal testing and therapeutic application, the survival of ISP was examined at different temperatures and in the complete pH range. Phage ISP was shown to be stable at 16°C, 37°C and 42°C, while freezing at −20°C and heating to 50°C reduced the phage titer with more than two logarithmic units (Figure S2 (A)). Consequently, ISP should be suited for the treatment of humans and animals, including those with fever, and the decontamination of hospital and farm environments. At pronounced acid (pH 1–3) or alkaline (pH 11–13) conditions, ISP is completely inactivated (Figure S2 (B)). A pH of 4 resulted in a significant logarithmic reduction of the titer, while ISP was stable between pH 5 and 9. This implies that ISP will not survive in gastric acid (pH 1–2) and therefore cannot be administered orally without manipulation. Blood has a neutral pH and might thus provide a suitable environment for phages. To exclude any negative effects of blood components on the viability of phages in an *ex vivo* situation, ISP was incubated in rabbit blood which was collected using a syringe rinsed with heparin and transported in blood collection tubes (Microvette 500 Lithium Heparin, SARSTEDT AG & Co., Nümbrecht, Germany) containing heparin to prevent blood clotting. No loss of infectivity was observed, enabling potential intravenous phage administration.

In a preliminary study, the safety of ISP application was assessed in hybrid albino rabbits following approval of the ethical committee from the Faculty of Veterinary Medicine of Ghent University (EC 2008_112, EC 2009_063). Nasal administration in both nasal cavities as well as subcutaneous injection (1 ml syringe and 26 G×1/23″ needle, Terumo International, Leuven, Belgium) of 10^9^ ISP particles was evaluated in three rabbits. In a parallel experiment, three rabbits first received 3 mg/kg ranitidine (Zantac®, Glaxo Smith Kline, Genval, Belgium) to inhibit gastric acid secretion, followed by an oral dose of 10^9^ ISP particles. Subsequent daily clinical observation of the rabbits for at least 5 days revealed no adverse effects: the rabbits had a healthy appetite, a normal rectal temperature and general condition and no symptoms of illness. For ISP detection purposes after oral administration, blood samples were obtained from the lateral ear vein at several time points from 5 minutes till 12 hours after phage administration using a 24 G×3/4″ catheter (Terumo International). Following euthanasia with 0.5 ml/kg T61® (Intervet, Brussels, Belgium), the faeces were collected and tissue samples were taken from the liver, kidneys and spleen. ISP could not be detected in the blood, the organs or the faeces of the rabbits.

### Analysis of the ISP genome

**Figure 3:**
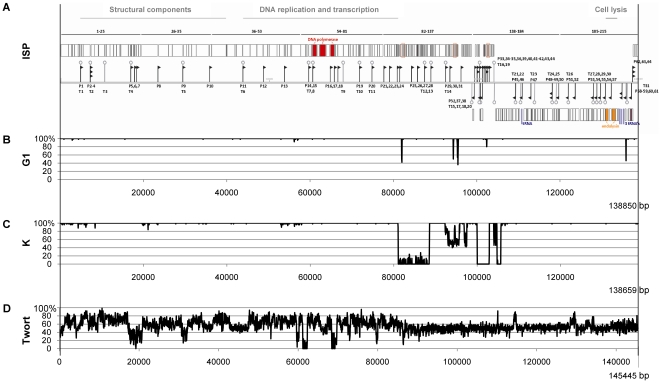
Summary of the bio-informatical analysis of the phage ISP genome. (A) Representation of the genome of phage ISP. Predicted proteins are indicated as bars, putative promoters are depicted as arrows and putative terminators as hair pins and the 4 tRNA's are indicated in light blue. The DNA polymerase and the endolysin, both interrupted by endonucleases, are colored red and orange respectively. Genes which were not annotated in phage G1 are colored purple and genes mistakenly annotated in phage G1 in dark blue, additional genes in phage ISP are framed in red. (B, C and D) Pairwise DNA homology of ISP and G1, ISP and K and ISP and Twort, compared using a sliding window of 50 bp.

ISP DNA was extracted from a CsCl-purified stock solution (>10^9^ pfu/ml) according to Sambrook and Russell [3]. Initial sequencing was performed by shotgun sequencing of a library of phage DNA in a pUC18 vector (Fermentas GmbH, St. Leon-Rot, Germany) combined with primer walking until a single contig was obtained. The linear double-stranded DNA of ISP with terminally redundant ends and containing 138.339 bp (EMBL: FR852584) was sequenced with an average redundancy of 6.7 and a coverage of each base position with at least three sequencing reactions. ORF Finder (http://www.ncbi.nlm.nih.gov/projects/gorf/), GeneMark™ [16] and BLASTx [17] were used to predict 215 open reading frames (ORFs) (Figure 3), followed by manual verification. Further, the predicted protein sequences were compared with known proteins and putative functions were assigned using BLASTp [17] and HHpred [18]. The nucleotide positions and additional information of all predicted genes are shown in Table S3. Finally, four tRNA's, encoding Asp-tRNA, Phe-tRNA, Trp-tRNA and Met-tRNA, were predicted in two non-coding regions of the genome using tRNAscan-SE [19] and ARAGORN [20].

K [21], G1 and Twort [2] are the best-known representatives of the ‘Twort-like viruses’ [22], myoviruses infecting gram-positive, low G+C content bacteria, and are assumed to show promising therapeutic potential. DNA homology with these phages was examined with EMBOSS stretcher [23], [24]. The genome of ISP is 99.5% identical to the 138.715 bp genome of phage G1 (Figure 3). The mutual differences comprise small sequence deletions and insertions which result in three extra hypothetical genes in ISP: ORF92, ORF126 and ORF147. Seven genes of G1 were chosen not to be annotated (ORF216, ORF247, ORF285, ORF309, ORF311, ORF388 and ORF450), due to their gene location integral in other genes or their questionable or absent Shine-Dalgarno sequence. In contrast, five additional genes which were not annotated in G1, ORF89, ORF103, ORF104, ORF140 and ORF205, were added to the protein-coding part of the ISP genome. At the nucleotide level, ISP displays 90.6% and 56.1% homology with K (127.395 bp) and Twort (130.706 bp) respectively, as shown in Figure 3. The differences with K are mainly confined to a region of 25 kb containing only hypothetical genes, while the variation between ISP and Twort is found throughout their genomes. An overview of their homologous genes is presented in Figure S3. In addition, several ISP genes were found to be homologous with predicted genes of *Listeria* phage A511 and *Enterococcus* phage phiEF24C, a ‘Twort-like virus’ and an orphan of the *Spounavirinae*, respectively [22].

As described earlier for the phages K [21] and Twort [2], ISP has its genes organized in three major modules encoding structural proteins, components of the replication and transcription machinery, and proteins responsible for cell lysis. Further, these phages are typified by an overall conserved gene organization, insertions and deletions, and the presence of unrelated genes between conserved genes [2]. For the purpose of phage therapy, the ISP genome encodes no potential gene products resembling known virulence or toxic proteins.

Phage ISP does not encode an RNA polymerase, implying its dependency on the host RNA polymerase for transcription. In this context, phage ISP encodes a sigma factor-binding polypeptide, Gp47, first identified in phage G1 by Dehbi *et al.* [25]. This polypeptide, which displays a growth-inhibitory effect when overexpressed in *S. aureus*, is considered to be one of the crucial components in the phage strategy to redirect the host RNA polymerase to transcription of phage DNA.

Conserved regulatory motifs in the ISP genome were searched using Nostradamus [26], PHIRE [27] and MEME/MAST [28] and resulted in the prediction of 64 host-specific promoters (Table S4). These putative promoters contain a variety of conserved motifs and a spacer region with a length of 17 or 18 nucleotides. The −35 box sequence is well-conserved, whereas more sequence variation is observed in the −10 box. Both the −35 box and the −10 box comprise six nucleotides and their conservation is graphically analyzed with WebLogo [29] (Figure S4). In addition, Palindrome [23] was used to detect 31 potential factor-independent terminators, while the free energy of their secondary structures was computed with Mfold [30] (Table S5). Both the promoters and the terminators are equally distributed over the ISP genome.

### Proteome analysis of the ISP particle

**Figure 4:**
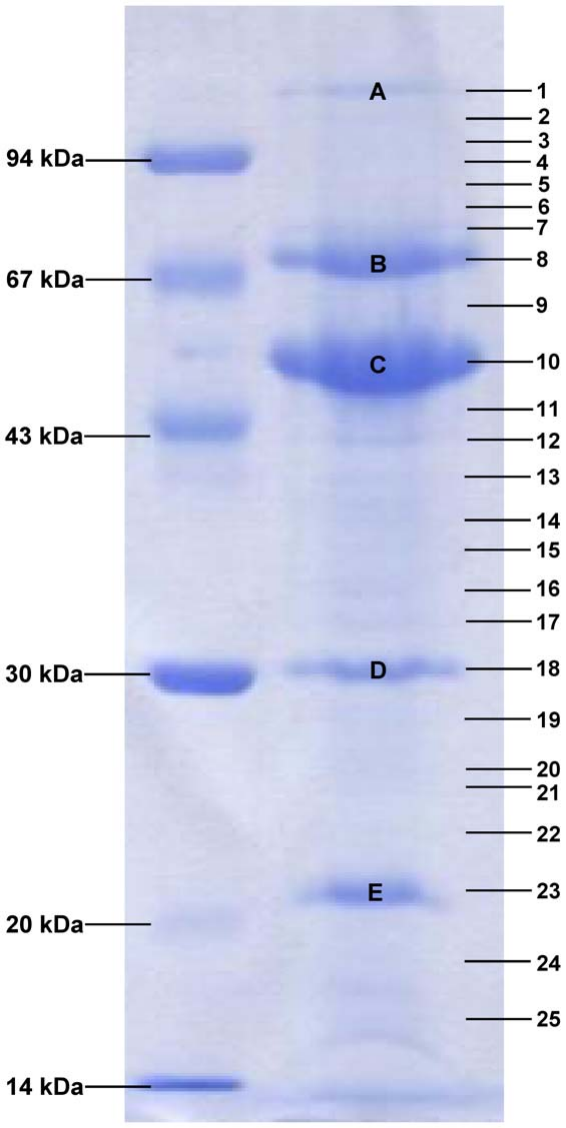
Coomassie G-250 stained SDS-PAGE gel of the structural proteome of phage ISP. On the left a low molecular weight marker is shown. The numbers on the right correspond to the analyzed gel pieces of which the identified proteins are given in Table 1. Clearly visible protein bands are indicated with capital letters.

In a next step, electrophoretic and mass spectrometric techniques were used to elucidate the composition of the ISP virion. Extraction of phage proteins from a CsCl-purified ISP stock solution (>10^9^ pfu/ml) was performed by methanol/chloroform extraction (1∶1∶0.75, v/v/v). Following gel electrophoresis, five major protein bands were clearly visualized with Simply Blue™ Safe Stain (Invitrogen Ltd, Paisley, UK) (Figure 4). Nonetheless the whole SDS-PAGE gel was fragmented and subjected to tandem electrospray ionization-mass spectrometry (ESI-MS/MS) on a LCQ Classic (ThermoFinnigan) equipped with a nano-LC column switching system as described by Lavigne *et al.* [31]. In 25 gel pieces, peptides of 22 phage proteins were identified (Table 1). Thirteen of these proteins were already listed as virion components and the structural function of Gp30, Gp38, Gp40, Gp72 and Gp186 was confirmed by Eyer *et al.* in 2007 [32]. Six additional proteins, Gp2, Gp13, Gp16, Gp28, Gp31 and Gp83, can be conferred to the group of structural proteins. However, nine proteins, identified by Eyer *et al.* [32] using one-dimensional and two-dimensional approaches, were not found in the present study. Generally, the identified proteins were localized in a major (Gp8–Gp40) and a minor (Gp70–Gp72) structural region in the ISP genome.

**Table 1:** Characteristics of the phage ISP ESI-MS/MS identified proteins.

ORF	Band number	Molecular mass (kDa)	Number of identified peptides	Sequence coverage (%)	HHpred search	e-value	K/G1 homologue^1^	Identification method^1^
Gp2	19	30.6	1	8.4			/	/
Gp8	8	64.0	12	27.4	Portal protein (HK97)	6.10E-41	ORF 41 (K)	2-DE
Gp11	1, 2, 4–13, 15–25	51.2	15	63.9	Capsid protein (HK97)	5.60E-01	ORF 44 (K)	SDS-PAGE, 2-DE
Gp13	14	34.1	3	12.9	Tail fiber protein	1.60E+02	/	/
Gp16	16	31.8	4	21.9	Baseplate hub assembly protein (T4)	1.50E+02	/	/
Gp18	1, 2, 4–11, 13, 15–25	64.4	21	62.7	Tail sheath protein (T4)	0	ORF 49 (K)	SDS-PAGE, 2-DE
Gp19	1, 22–25	15.9	5	54.2	Tail tube protein (T4)	1.00E+01	ORF 50 (K)	SDS-PAGE, 2-DE
Gp26	1	143.9	1	1.4	Fibritin (T4)	3.10E-04	ORF 55 (K)	SDS-PAGE
Gp28	15	34.6	2	10.5	Peptidoglycan hydrolase (Dp-1)	4.60E-11	/	/
Gp30	16	29.3	4	24.3			ORF 59 (K)	2-DE
Gp31	21	19.9	1	6.3			/	/
Gp32	16	26.6	4	25.6	Baseplate wedge subunit (T4)	2.00E-19	/	/
Gp33	10, 15	39.1	3	6.9	Baseplate J-like protein (P2)	3.90E-42	ORF 62 (K)	SDS-PAGE, 2-DE
Gp35	21, 22, 25	19.2	5	44.5	Baseplate structural protein (T4)	3.10E-04	ORF 64 (K)	SDS-PAGE, 2-DE
Gp36	1–8, 15, 17, 24	129.1	33	40.5			ORF 65 (K)	SDS-PAGE, 2-DE
Gp38	7, 8, 15–17	72.6	20	41.7			ORF 66 (K)	2-DE
Gp40	17	50.4	2	7.0			ORF 68 (K)	SDS-PAGE, 2-DE
Gp70	1, 15–25	23.2	9	67.1	Major tail protein V (Lambda)	5.20E+01	ORF 95 (K)	SDS-PAGE, 2-DE
Gp71	18, 21	17.8	3	42.4	Major tail protein V (Lambda)	4.60E-21	ORF 96 (K)	SDS-PAGE, 2-DE
Gp72	25	7.8	2	28.0			ORF 189 (G1)	2-DE
Gp83	23	17.8	1	7.9			/	/
Gp186	25	10.1	1	17.2			ORF 172 (G1)	2-DE

1 Adjusted from Eyer *et al.* (2007).

For each gene product (Gp) the band number(s) (according to Figure 4), the molecular weight in kDa, the number of identified peptides (>99% protein identification probability with manual validation), the protein sequence coverage in percent, the putative protein function according to HHpred and the corresponding e-value are shown. Further, earlier identified homologous proteins of phage K or G1 and the corresponding identification method (SDS-PAGE/2-DE) are listed.

Several ISP proteins were found throughout the entire SDS-PAGE gel, illustrating their abundance in the phage virion. The major capsid protein (Gp11), most likely present in the most prominent protein band (C), exemplifies this phenomenon. The abundant presence of these proteins may have hindered the identification of less abundant structural proteins. According to the molecular mass, band A and E contain Gp26 and Gp70 respectively. The band B protein (approximately 70 kDa) is indefinable because both the molecular mass of Gp18 (64.4 kDa) and Gp38 (72.6 kDa) approach its position. In protein band D (30 kDa) no protein with a corresponding molecular mass was found, but this band may represent the modified form of the tail sheath protein (Gp18) linked to tail contraction as reported by Chibani-Chennoufi *et al.* [33] and Eyer *et al.* [32].

To support the mass spectrometric identification of the 22 proteins in this analysis, a more profound database search using HHpred was performed, in which a higher e-value was also taken into account (Table 1). Interestingly, ISP virion proteins are related to myoviral as well as siphoviral structural components. Various identified ISP proteins show homology with structural proteins of the *E. coli* phage T4. The contractile tail of this myovirus consists of a contractile sheath, a tail tube and a baseplate [34]. Gp16 is similar to a T4 protein responsible for the formation of the central hub of the baseplate and Gp32 and Gp35 are homologous to structural components of the T4 baseplate. Gp18 and Gp19 are related to the tail sheath protein and the tail tube protein of phage T4, whereas Gp26 resembles fibritin which attaches the tail fibers to the T4 particle. Five other structural ISP proteins show homology with temperate *E. coli* phages. The portal protein and capsid protein of ISP are related to those of phage HK97, while Gp70 and Gp71 show similarity to the major tail protein of phage Lambda and Gp33 is similar to the baseplate protein J of phage P2. Finally, Gp13 and Gp28 were examined, revealing homology with the peptidoglycan hydrolase of the pneumococcal phage Dp-1 and a phage tail fiber domain, respectively. These findings support the hypothesis of Chibani-Chennoufi *et al.* that the SPO1-related viruses share structural homology with *Siphoviridae* more than with other *Myoviridae* [33].

## Discussion

By describing the composition, laboratory-based production and quality control of an experimental phage cocktail containing *S. aureus* phage ISP, Merabishvili *et al.* [1] support the practical re-introduction of phage therapy in Western medicine. The microbiological and molecular characterization of phage ISP in the present report is a necessary step in the medical exploitation of the antibacterial potential of BFC-1. The presented host range screening including clinically relevant MRSA strains, the determination of the infection parameters and the assessment of the ISP virion stability (temperature, pH, blood components) contribute to the further development of the therapeutic application of ISP. Moreover, phage ISP can be safely administered orally, in the nose and through subcutaneous injection. In case of oral application, ISP did not reach the blood, the faeces or the organs. In addition, the genome analysis of ISP, which revealed no toxic or virulence-associated genes, emphasizes the mutual similarity between the ‘Twort-like viruses’, which remains the only known genus of virulent *S. aureus*-infecting viruses so far.

## Supporting Information

Figure S1 **Adsorption curve of phage ISP on** ***S. aureus*** **subsp.** ***aureus*** **Rosenbach ATCC 6538.** The proportion of the amount of non-adsorbed phages to the amount of phages used for infection, based on three independent experiments, is shown and standard deviations are indicated.
(TIF)

Figure S2 **Biophysical stability of phage ISP.** The logarithmic drop in infectious ISP particles after 24 hours of incubation at different pH levels (A) and at different temperatures (B) is shown. Three independent experiments were performed, standard deviations are indicated.
(TIF)

Figure S3 **Summary of the common genes of the phages ISP, K and Twort.** The genome of ISP contains 83 genes which have homologous counterparts in K and Twort. Further, ISP shares 85 and 1 additional homologous genes with K and Twort repectively. In contrast, K and Twort have no additional common genes. Forty-one ISP genes absent in phage K are generally organized in three major blocks and situated downstream the DNA replication and transcription module. The differences between ISP and Twort are located througout their whole genome sequence. ISP and Twort have 41 and 109 unique genes respectively and their common genes are about 70% homologous.
(TIF)

Figure S4 **Sequence logo of the conserved motifs of the promoters of phage ISP.** The conservation of the −35 box (A) and the −10 box (B) of the 65 promoters predicted in the genome of ISP is depicted. The height of each stack designates the sequence conservation at that position (measured in bits), while the height of the symbols within each stack indicates the relative frequency of the nucleotide at that position.
(TIF)

Table S1 **Overview of the host range of** ***Staphylococcus*** **phage ISP.** The clarity of the lysis zones after spotting several phage dilutions was scored as clear and turbid and the dilution on which lysis spots and individual plaques appeared was taken into account. These observations in combination with a verifying plaque assay were used to differentiate between sensitive (+) and insensitive (−) to ISP infection on one hand and a low, moderate and high efficiency of plating on the other hand. For each isolate, the species, the origin, the sensitivity to ISP infection and the efficiency of plating is given. For 33 isolates the presence (+) or absence (−) of genes encoding resistance to methicillin, MLS^3^ antimicrobials, aminoglycosides and tetracycline, as well as the presence or absence of penicillinase is shown.
(DOCX)

Table S2 **Mutation rate for ISP resistance.** For five human *S. aureus* isolates, the mutation rate conferring ISP resistance was calculated by dividing the number of resistant colonies by the number of bacterial cells at the time of ISP application. Based on five individual experiments, the mean values and corresponding standard deviations are indicated.
(DOCX)

Table S3 **Features of the predicted ORFs of phage ISP.** For each predicted ORF the start and stop position in the genome, the length of the corresponding gene product in amino acids, the reading frame, the start and stop codon, the putative protein function and the corresponding prediction program and e value are shown.
(DOCX)

Table S4 **Predicted host-specific promotors of phage ISP.** For each promotor the strand, the start and stop position in the genome, the −35 box, the spacer region, the −10 box and the length of the spacer region are given.
(DOCX)

Table S5 **Predicted factor-independent terminators of phage ISP.** For each terminator the strand, the start and stop position in the genome, the free energy of their secundary structure and the sequence of the regulatory element (the palindromic sequence is underlined) are given.
(DOCX)

## References

[1] Merabishvili M, Pirnay J-P, Verbeken G, Chanishvili N, Tediashvili L (2009). Quality-controlled small-scale production of a well-defined bacteriophage cocktail for use in human clinical trials.. PLoS ONE.

[2] Kwan T, Liu J, DuBow M, Gros P, Pelletier J (2005). The complete genomes and proteomes of 27 *Staphylococcus aureus* bacteriophages.. PNAS.

[3] Sambrook J, Russell DW (2001). Molecular Cloning: A Laboratory Manual, 3rd ed.

[4] Monecke S, Jatzwauk L, Weber S, Slickers P, Ehricht R (2008). DNA microarray-based genotyping of methicillin-resistant *Staphylococcus aureus* strains from Eastern Saxony.. Clin Microbiol Infect.

[5] Monecke S, Coombs G, Shore AC, Coleman DC, Akpaka P (2011). A Field Guide to Pandemic, Epidemic and Sporadic Clones of Methicillin-Resistant *Staphylococcus aureus*.. PLoS One.

[6] Vanderhaeghen W, Hermans K, Haesebrouck F, Butaye P (2010). Methicillin-Resistant *Staphylococcus aureus* (MRSA) in Food Production Animals.. Epidemiol Infec.

[7] Smith TC, Pearson N (2011). The Emergence of *Staphylococcus aureus* ST398.. Vector Borne Zoonotic Dis.

[8] Adams MH (1959). Bacteriophages.

[9] Ceyssens P-J, Noben J-P, Ackermann HW, Verhaegen J, De Vos D (2009). Survey of *Pseudomonas aeruginosa* and its phages: de novo peptide sequencing as a novel tool to assess the diversity of worldwide collected viruses.. Environ Microbiol.

[10] Ceyssens P-J, Lavigne R, Mattheus W, Chibeu A, Hertveldt K (2006). Genomic Analysis of *Pseudomonas aeruginosa* Phages LKD16 and LKA1: Establishment of the phiKMV Subgroup within the T7 Supergroup.. J Bacteriol.

[11] Beale GH (1949). A Method for the Measurement of Mutation Rate from Phage Sensitivity to Phage Resistance in *Escherichia coli*.. Microbiol.

[12] Chibeu A, Ceyssens P-J, Hertveldt K, Volckaert G, Cornelis P (2009). The adsorption of *Pseudomonas aeruginosa* bacteriophage phiKMV is dependent on expression regulation of type IV pili genes.. FEMS Microbiol Let.

[13] Donadio S, Paladino R, Costanzi I, Sparapani P, Schreil W (1986). Characterization of Bacteriophages Infecting *Streptomyces erythreus*.. J Bacteriol.

[14] Binettia AG, Bailoa NB, Reinheimer JA (2007). Spontaneous phage-resistant mutants of *Streptococcus thermophilus*: Isolation and technological characteristics.. Int Diary J.

[15] Sulakvelidze A, Kutter E, Kutter E, Sulakvelidze A (2005). Bacteriophage Therapy in Humans.. Bacteriophages: Biology and Applications.

[16] Lukashin AV, Borodovsky M (1998). GeneMarkhmm: new solutions for gene finding.. Nucleic Acids Res.

[17] Altschul SF, Gish W, Miller W, Myers EW, Lipman DJ (1990). Basic local alignment search tool.. J Mol Biol.

[18] Söding J, Biegert A, Lupas AN (2005). The HHpred interactive server for protein homology detection and structure prediction.. Nucleic Acids Res.

[19] Lowe TM, Eddy SR (1997). tRNAscan-SE: a program for improved detection of transfer RNA genes in genomic sequence.. Nucleic Acids Res.

[20] Laslett D, Canback B (2004). ARAGORN, a program to detect tRNA genes and tmRNA genes in nucleotide sequences.. Nucleic Acids Res.

[21] O'Flaherty S, Coffey A, Edwards R, Meaney W, Fitzgerald GF (2004). Genome of Staphylococcal Phage K: a New Lineage of *Myovirdae* Infecting Gram-Positive Bacteria with a Low G+C Content.. J Bacteriol.

[22] Lavigne R, Darius P, Summer EJ, Seto D, Mahadevan P (2009). Classification of *Myoviridae* bacteriophages using protein sequence similarity.. BMC Microbiol.

[23] Myers E, Miller W (1988). “Optimal Alignments in Linear Space”.. CABIOS.

[24] Rice P, Longden I, Bleasby A (2000). EMBOSS: the European molecular biology open software suite.. Trends Genet.

[25] Dehbi M, Moeck G, Arhin FF, Bauda P, Bergeron D (2009). Inhibition of Transcription in *Staphylococcus aureus* by a Primary Sigma Factor-Binding Polypeptide from Phage G1.. J Bacteriol.

[26] Gordon L, Chervonenkis AY, Gammerman AJ, Ilham A, Shahmuradov IA (2003). “Sequence Alignment Kernel for recognition of promoter regions”.. Bioinform.

[27] Lavigne R, Sun W, Volckaert G (2004). PHIRE, a deterministic approach to reveal regulatory elements in bacteriophage genomes.. Bioinform.

[28] Bailey TL, Elkan C (1994). “Fitting a mixture model by expectation maximization to discover motifs in biopolymers”.. Proc Int Conf Intell Syst Mol Biol.

[29] Crooks GE, Hon G, Chandonia J-M, Brenner SE (2004). WebLogo: A Sequence Logo Generator.. Genome Res.

[30] Zuker M (2003). Mfold web server for nucleic acid folding and hybridization prediction.. Nucleic Acids Res.

[31] Lavigne R, Noben JP, Hertveldt K, Ceyssens P-J, Briers Y (2006). The structural proteome of *Pseudomonas aeruginosa* bacteriophage phiKMV.. Microbiology.

[32] Eyer L, Pantůček R, Zdráhal Z, Konečná H, Kašpárek P (2007). Structural protein analysis of the polyvalent staphylococcal bacteriophage 812.. Proteomics.

[33] Chibani-Chennoufi S, Dillmann M-L, Marvin-Guy L, Rami-Shojaei S, Brüssow H (2004). *Lactobacillus plantarum* bacteriophage LP65: a new member of the SPO1-like genus of the family *Myoviridae*.. J Bacteriol.

[34] Miller ES, Kutter E, Mosig G, Arisaka F, Kunisawa T (2003). Bacteriophage T4 genome.. Microbiol Mol Biol R.

