# Supplementary figures and images for: Microbiological and Molecular Assessment of Bacteriophage ISP for the Control of Staphylococcus aureus
Source: PLoS One. 2011 Sep 9;6(9):e24418. doi: 10.1371/journal.pone.0024418 (PMC3170307; doi:10.1371/journal.pone.0024418)

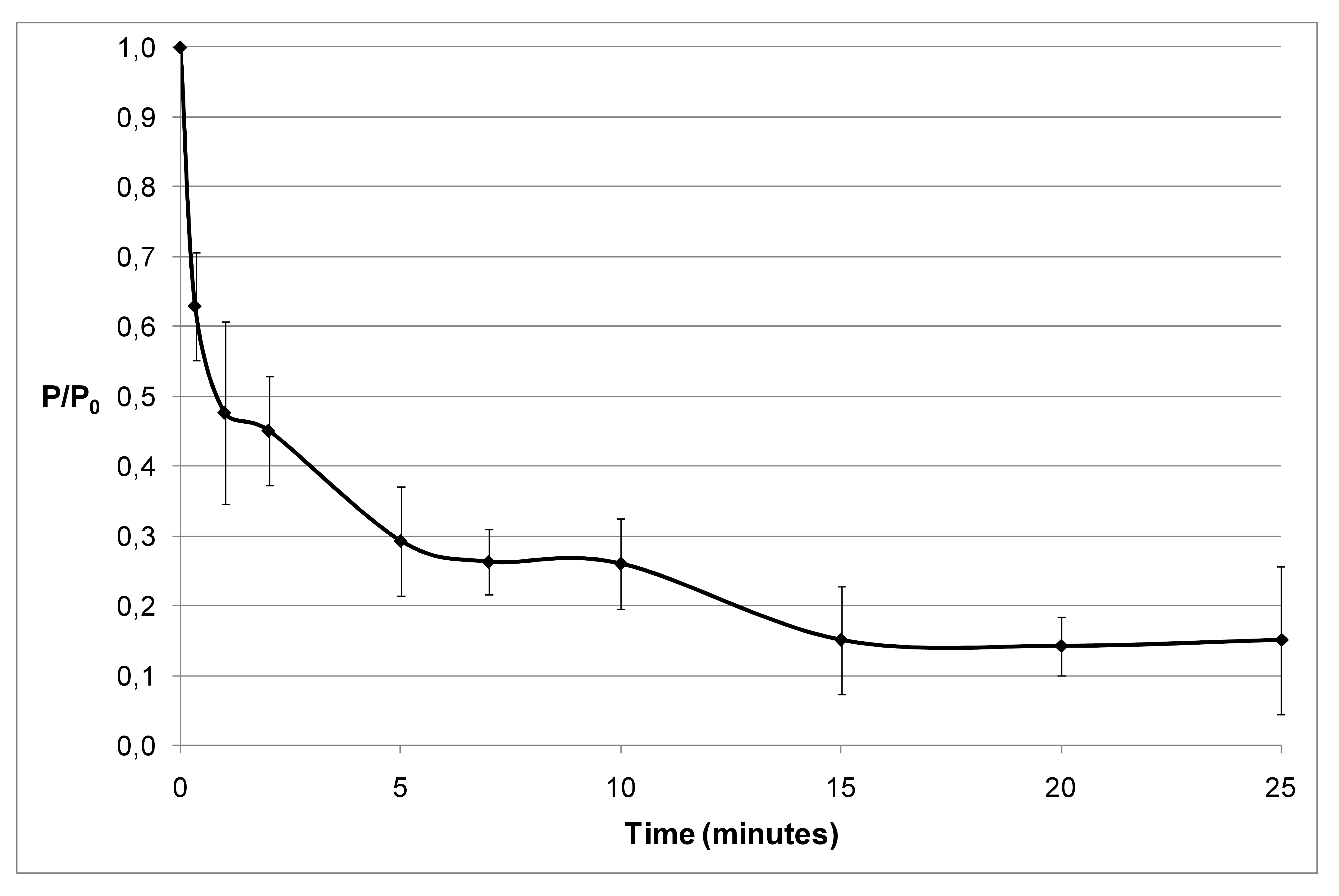

Supplement: Figure S1 — Adsorption curve of phage ISP on S. aureus subsp. aureus Rosenbach ATCC 6538. The proportion of the amount of non-adsorbed phages to the amount of phages used for infection, based on three independent experiments, is shown and standard deviations are indicated. (TIF) [file pone.0024418.s001.tif]

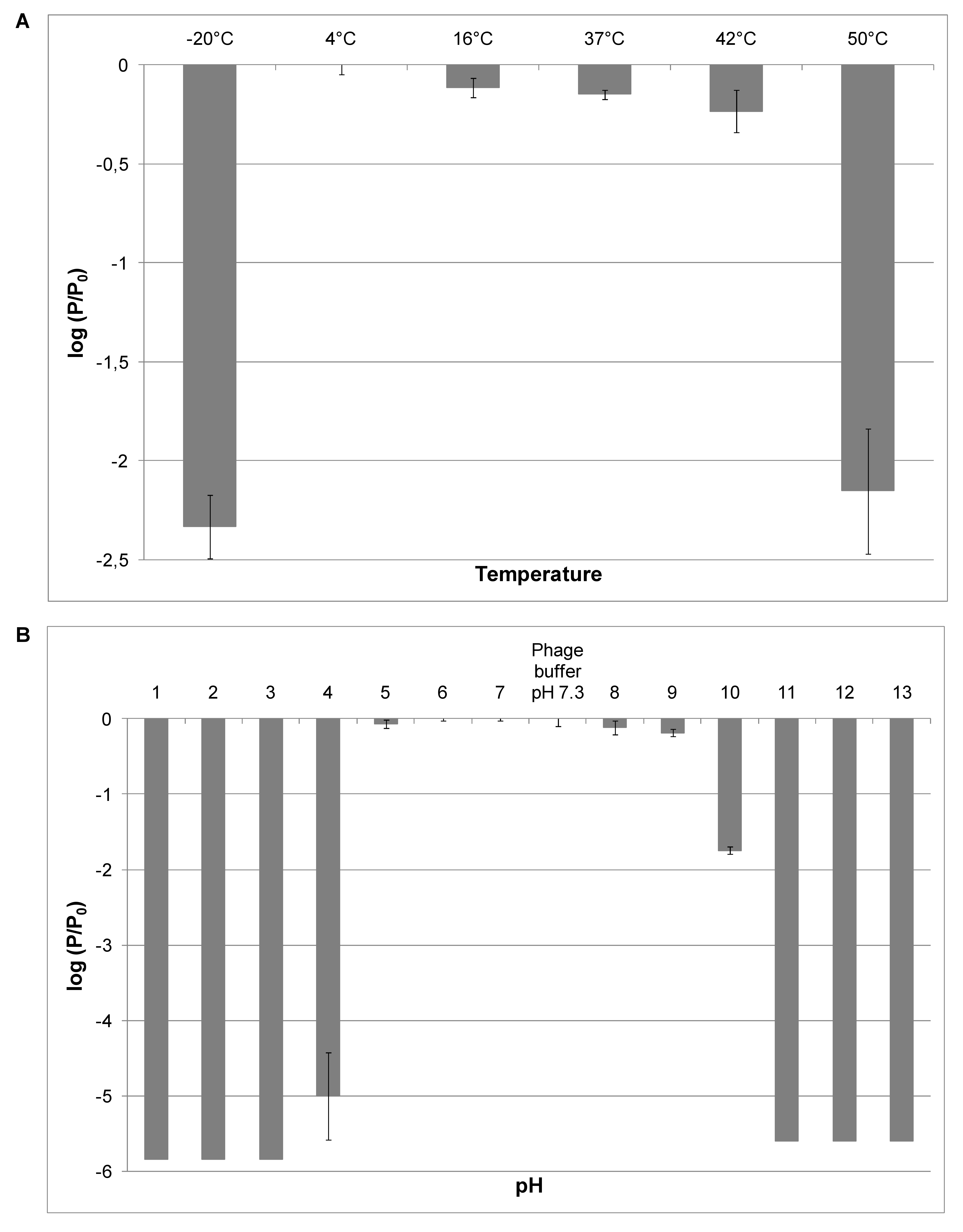

Supplement: Figure S2 — Biophysical stability of phage ISP. The logarithmic drop in infectious ISP particles after 24 hours of incubation at different pH levels (A) and at different temperatures (B) is shown. Three independent experiments were performed, standard deviations are indicated. (TIF) [file pone.0024418.s002.tif]

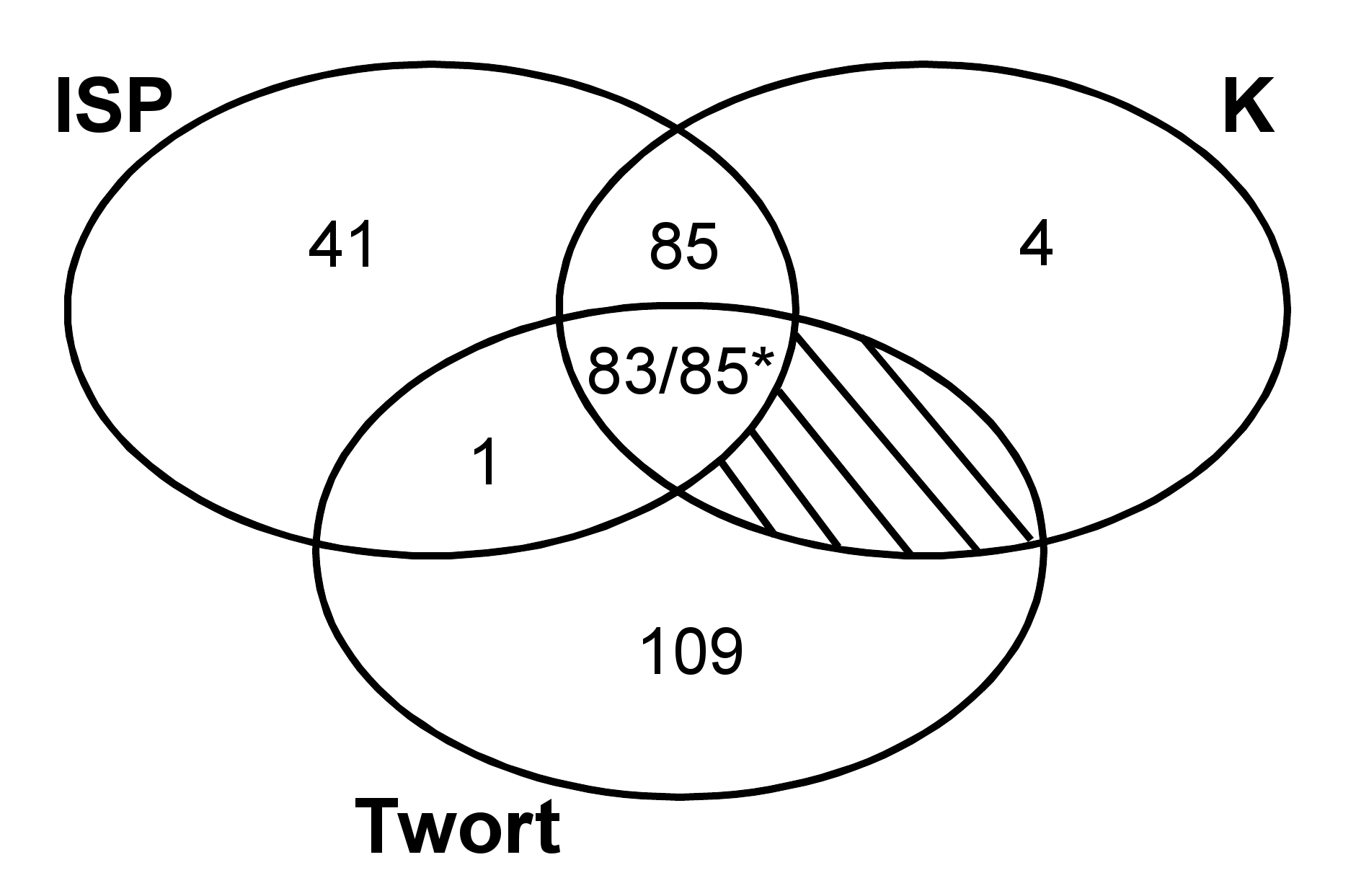

Supplement: Figure S3 — Summary of the common genes of the phages ISP, K and Twort. The genome of ISP contains 83 genes which have homologous counterparts in K and Twort. Further, ISP shares 85 and 1 additional homologous genes with K and Twort repectively. In contrast, K and Twort have no additional common genes. Forty-one ISP genes absent in phage K are generally organized in three major blocks and situated downstream the DNA replication and transcription module. The differences between ISP and Twort are located througout their whole genome sequence. ISP and Twort have 41 and 109 unique genes respectively and their common genes are about 70% homologous. (TIF) [file pone.0024418.s003.tif]

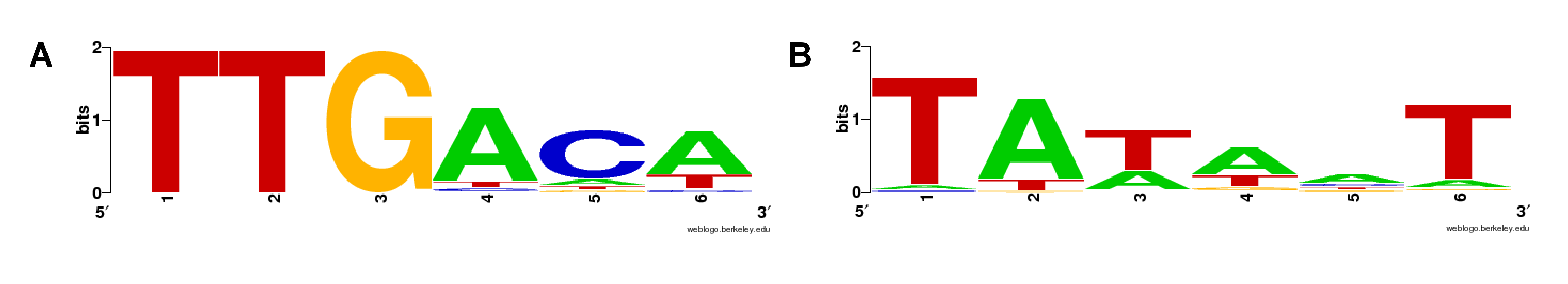

Supplement: Figure S4 — Sequence logo of the conserved motifs of the promoters of phage ISP. The conservation of the −35 box (A) and the −10 box (B) of the 65 promoters predicted in the genome of ISP is depicted. The height of each stack designates the sequence conservation at that position (measured in bits), while the height of the symbols within each stack indicates the relative frequency of the nucleotide at that position. (TIF) [file pone.0024418.s004.tif]
