# Supplementary material for: Microbiological and Molecular Assessment of Bacteriophage ISP for the Control of Staphylococcus aureus
Source: PLoS One. 2011 Sep 9;6(9):e24418. doi: 10.1371/journal.pone.0024418 (PMC3170307; doi:10.1371/journal.pone.0024418)
Supplement: Table S1 — Overview of the host range of Staphylococcus phage ISP. The clarity of the lysis zones after spotting several phage dilutions was scored as clear and turbid and the dilution on which lysis spots and individual plaques appeared was taken into account. These observations in combination with a verifying plaque assay were used to differentiate between sensitive (+) and insensitive (−) to ISP infection on one hand and a low, moderate and high efficiency of plating on the other hand. For each isolate, the species, the origin, the sensitivity to ISP infection and the efficiency of plating is given. For 33 isolates the presence (+) or absence (−) of genes encoding resistance to methicillin, MLS3 antimicrobials, aminoglycosides and tetracycline, as well as the presence or absence of penicillinase is shown. (DOCX) [file pone.0024418.s005.docx]

| **Strain** | **Species** | **Origin** | **Methicillin** | **Penicillinase** | **MLS^3^** | **Aminoglycosides** | **Tetracycline** | **ISP infectivity** | **Efficiency of plating** |
| --- | --- | --- | --- | --- | --- | --- | --- | --- | --- |
| PS^1^ 3A | *S. aureus* | / | / | / | / | / | / | + | High |
| PS3C | *S. aureus* | / | / | / | / | / | / | + | High |
| PS6 | *S. aureus* | / | / | / | / | / | / | + | High |
| PS29 | *S. aureus* | / | / | / | / | / | / | + | High |
| PS42D | *S. aureus* | / | / | / | / | / | / | + | High |
| PS42E | *S. aureus* | / | / | / | / | / | / | + | High |
| PS47 | *S. aureus* | / | / | / | / | / | / | + | High |
| PS52 | *S. aureus* | / | / | / | / | / | / | + | Moderate |
| PS52A/79 | *S. aureus* | / | / | / | / | / | / | + | Moderate |
| PS53 | *S. aureus* | / | / | / | / | / | / | + | High |
| PS54 | *S. aureus* | / | / | / | / | / | / | + | High |
| PS55 | *S. aureus* | / | / | / | / | / | / | + | High |
| PS71 | *S. aureus* | / | / | / | / | / | / | + | High |
| PS75 | *S. aureus* | / | / | / | / | / | / | + | High |
| PS77 | *S. aureus* | / | / | / | / | / | / | - | / |
| PS80 | *S. aureus* | / | / | / | / | / | / | + | Moderate |
| PS81 | *S. aureus* | / | / | / | / | / | / | + | High |
| PS83A | *S. aureus* | / | / | / | / | / | / | + | High |
| PS84 | *S. aureus* | / | / | / | / | / | / | + | Moderate |
| PS85 | *S. aureus* | / | / | / | / | / | / | - | / |
| PS88 | *S. aureus* | / | / | / | / | / | / | - | / |
| PS89 | *S. aureus* | / | / | / | / | / | / | + | High |
| PS90 | *S. aureus* | / | / | / | / | / | / | - | / |
| PS92 | *S. aureus* | / | / | / | / | / | / | + | High |
| PS94 | *S. aureus* | / | / | / | / | / | / | - | / |
| PS95 | *S. aureus* | / | / | / | / | / | / | - | / |
| PS96 | *S. aureus* | / | / | / | / | / | / | - | / |
| PS187 | *S. aureus* | / | / | / | / | / | / | + | Moderate |
| PSD11 | *S. aureus* | / | / | / | / | / | / | - | / |
| PSHK2 | *S. aureus* | / | / | / | / | / | / | - | / |
| PSPB | *S. aureus* | / | / | / | / | / | / | + | Moderate |
| KS^2^ 1 | *S. aureus* | Patient (UZ Leuven) | + | + | - | - | + | + | High |
| KS2 | *S. aureus* | Patient (UZ Leuven) | + | + | + | - | + | + | High |
| KS3 | *S. aureus* | Patient (UZ Leuven) | + | - | + | - | + | + | High |
| KS4 | *S. aureus* | Patient (UZ Leuven) | + | + | - | - | + | + | High |
| KS5 | *S. aureus* | Patient (UZ Leuven) | + | + | - | - | + | + | High |
| KS6 | *S. aureus* | Patient (UZ Leuven) | + | + | + | + | + | + | High |
| KS7 | *S. aureus* | Patient (UZ Leuven) | + | + | + | + | + | + | High |
| KS8 | *S. aureus* | Patient (UZ Leuven) | + | + | - | + | + | + | High |
| KS9 | *S. aureus* | Patient (UZ Leuven) | + | + | + | - | + | + | High |
| KS10 | *S. aureus* | Patient (UZ Leuven) | + | + | - | - | + | + | High |
| KS11 | *S. aureus* | Patient (UZ Leuven) | + | + | + | - | + | + | High |
| KS12 | *S. aureus* | Patient (UZ Leuven) | + | + | - | - | + | + | High |
| KS13 | *S. aureus* | Patient (UZ Leuven) | + | + | - | + | + | + | High |
| KS14 | *S. aureus* | Patient (UZ Leuven) | + | + | - | - | + | + | High |
| KS15 | *S. aureus* | Patient (UZ Leuven) | / | / | / | / | / | + | High |
| KS16 | *S. aureus* | Patient (UZ Leuven) | / | / | / | / | / | + | High |
| KS18 | *S. aureus* | Patient (UZ Leuven) | / | / | / | / | / | + | High |
| KS20 | *S. aureus* | Patient (UZ Leuven) | / | / | / | / | / | + | High |
| KS21 | *S. aureus* | Patient (UZ Leuven) | / | / | / | / | / | + | High |
| KS22 | *S. aureus* | Patient (UZ Leuven) | / | / | / | / | / | + | High |
| KS23 | *S. aureus* | Patient (UZ Leuven) | / | / | / | / | / | + | High |
| KS24 | *S. aureus* | Patient (UZ Leuven) | / | / | / | / | / | + | High |
| KS25 | *S. aureus* | Patient (UZ Leuven) | / | / | / | / | / | + | High |
| KS26 | *S. aureus* | Patient (UZ Leuven) | / | / | / | / | / | + | Moderate |
| KS27 | *S. aureus* | Patient (UZ Leuven) | / | / | / | / | / | + | High |
| KS28 | *S. aureus* | Patient (UZ Leuven) | / | / | / | / | / | + | High |
| KS30 | *S. aureus* | Patient (UZ Leuven) | / | / | / | / | / | + | High |
| UG1 | *S. haemolyticus* | Patient (Ghent University Hospital) | / | / | / | / | / | - | / |
| UG2 | *S. haemolyticus* | Patient (Ghent University Hospital) | / | / | / | / | / | - | / |
| UG3 | *S. haemolyticus* | Patient (Ghent University Hospital) | / | / | / | / | / | - | / |
| UG4 | *S. haemolyticus* | Patient (Ghent University Hospital) | / | / | / | / | / | - | / |
| UG5 | *S. haemolyticus* | Patient (Ghent University Hospital) | / | / | / | / | / | - | / |
| UG6 | *S. haemolyticus* | Patient (Ghent University Hospital) | / | / | / | / | / | - | / |
| UG7 | *S. aureus* | Patient (Ghent University Hospital) | / | / | / | / | / | + | High |
| UG8 | *S. haemolyticus* | Patient (Ghent University Hospital) | / | / | / | / | / | - | / |
| UG9 | *S. aureus* | Patient (Ghent University Hospital) | + | + | + | + | + | + | High |
| UG10 | *S. aureus* | Patient (Ghent University Hospital) | + | + | + | + | + | + | High |
| UG11 | *S. aureus* | Patient (Ghent University Hospital) | + | + | + | + | + | + | High |
| UG12 | *S. aureus* | Patient (Ghent University Hospital) | + | + | + | + | + | + | High |
| S53 | *S. aureus* | Poultry | + | + | - | + | + | + | High |
| S62 | *S. aureus* | Poultry | + | + | + | - | + | + | Moderate |
| S64 | *S. aureus* | Poultry | + | + | + | + | + | + | Low |
| S69 | *S. aureus* | Poultry | + | + | - | + | + | + | High |
| S92 | *S. aureus* | Poultry | + | + | + | - | + | + | Moderate |
| AV4 | *S. aureus* | Horse | + | + | - | + | + | + | Low |
| AV4' | *S. aureus* | Horse | + | + | - | + | + | + | Low |
| AV6 | *S. aureus* | Horse | + | + | - | - | + | + | Moderate |
| AV10 | *S. aureus* | Horse | + | + | - | + | + | + | Low |
| AV18 | *S. aureus* | Horse | + | + | + | + | + | + | Low |
| AV65 | *S. aureus* | Horse | + | + | - | + | + | + | Low |
| KH115 | *S. aureus* | Rabbit | / | / | / | / | / | + | High |
| KH171 | *S. aureus* | Rabbit | / | / | / | / | / | + | High |
| Sp8 | *S. aureus* | Rabbit | / | / | / | / | / | + | High |
| Sp10 | *S. aureus* | Rabbit | / | / | / | / | / | + | High |
| Sp17 | *S. aureus* | Rabbit | / | / | / | / | / | + | High |
| Sp18 | *S. aureus* | Rabbit | / | / | / | / | / | + | High |
| Sp23 | *S. aureus* | Rabbit | / | / | / | / | / | + | High |
| 796N | *S. aureus* | Pig | + | + | - | + | + | - | / |
| 823N | *S. aureus* | Pig | + | + | - | + | + | - | / |
| 830N | *S. aureus* | Pig | + | + | - | + | + | - | / |
| *S. aureus* ATCC 6538 | *S. aureus* | Patient | - | - | - | - | + | + | High |
| *S. haemolyticus* JCSC 1435 | *S. haemolyticus* | Patient (Juntendo Hospital) | / | / | / | / | / | - | / |
| Clinical *S. haemolyticus* strain | *S. haemolyticus* | Patient (UZ Leuven) | / | / | / | / | / | - | / |

^1^ Propagation strain

^2^ Clinical strain

^3^ Macrolide-lincosamide-streptogramin
