# Supplementary material for: Microbiological and Molecular Assessment of Bacteriophage ISP for the Control of Staphylococcus aureus
Source: PLoS One. 2011 Sep 9;6(9):e24418. doi: 10.1371/journal.pone.0024418 (PMC3170307; doi:10.1371/journal.pone.0024418)
Supplement: Table S2 — Mutation rate for ISP resistance. For five human S. aureus isolates, the mutation rate conferring ISP resistance was calculated by dividing the number of resistant colonies by the number of bacterial cells at the time of ISP application. Based on five individual experiments, the mean values and corresponding standard deviations are indicated. (DOCX) [file pone.0024418.s006.docx]

| **Strain** | **Number of resistant colonies** | **Bacterial count at time of ISP application** | | **Mutation rate** |
| --- | --- | --- | --- | --- |
|  |  | **. 10^8^ cfu** | | **/10^7^ cfu** |
| *S. aureus* ATCC6538 | 57 ± 13 | | 6.8 ± 3.0 | 1.1 ± 0.85 |
| KS1 | 280 ± 31 | | 5.6 ± 1.9 | 5.8 ± 2.6 |
| KS18 | 138 ± 13 | | 5.2 ±1.6 | 2.8 ± 0.8 |
| KS24 | 74 ± 17 | | 4.9 ± 1.9 | 1.6 ± 0.47 |
| UG9 | 20 ± 6 | | 8.1 ± 3.7 | 0.32 ± 0.21 |
