# Supplementary material for: Microbiological and Molecular Assessment of Bacteriophage ISP for the Control of Staphylococcus aureus
Source: PLoS One. 2011 Sep 9;6(9):e24418. doi: 10.1371/journal.pone.0024418 (PMC3170307; doi:10.1371/journal.pone.0024418)
Supplement: Table S3 — Features of the predicted ORFs of phage ISP. For each predicted ORF the start and stop position in the genome, the length of the corresponding gene product in amino acids, the reading frame, the start and stop codon, the putative protein function and the corresponding prediction program and e value are shown. (DOCX) [file pone.0024418.s007.docx]

| **ORF** | **Start** | **Stop** | **Length (aa)** | **Frame** | **Start codon** | **Stop codon** | **Putative function** | **Prediction program** | **e-value** |
| --- | --- | --- | --- | --- | --- | --- | --- | --- | --- |
| ORF1 | 13 | 1830 | 605 | +1 | ATG | TAG | Terminase large subunit *(Listeria* phage A511) | BLASTp | 0 |
| ORF2* | 1823 | 2644 | 273 | +2 | ATG | TAG |  |  |  |
| ORF3 | 2622 | 2804 | 60 | +3 | GTG | TGA |  |  |  |
| ORF4 | 2801 | 3280 | 159 | +2 | ATG | TAA |  |  |  |
| ORF5 | 3322 | 4521 | 399 | +1 | TTG | TAA |  |  |  |
| ORF5.1 | 4124 | 4492 | 122 | +2 | GTG | TAG |  |  |  |
| ORF6 | 4607 | 4948 | 113 | +2 | ATG | TAA |  |  |  |
| ORF7 | 4966 | 5337 | 123 | +1 | TTG | TAG |  |  |  |
| ORF8* | 5341 | 7032 | 563 | +1 | TTG | TAA | Portal protein (*Staphylococcus* phage K) | BLASTp | 0 |
| ORF9 | 7226 | 7999 | 257 | +2 | TTG | TAG | Minor capsid protein; prohead protease (*Listeria* phage A511) | BLASTp | 1.00E-68 |
| ORF10 | 8018 | 8974 | 318 | +2 | ATG | TAA |  |  |  |
| ORF11* | 9090 | 10481 | 463 | +3 | ATG | TAA | Major capsid protein (*Staphylococcus* phage K) | BLASTp | 0 |
| ORF12 | 10573 | 10869 | 98 | +1 | ATG | TAA |  |  |  |
| ORF13* | 10882 | 11790 | 302 | +1 | ATG | TAA |  |  |  |
| ORF14 | 11804 | 12682 | 292 | +2 | ATG | TAA |  |  |  |
| ORF15 | 12682 | 13302 | 206 | +1 | ATG | TAA |  |  |  |
| ORF16* | 13321 | 14157 | 278 | +1 | ATG | TAG |  |  |  |
| ORF17 | 14159 | 14374 | 71 | +2 | ATG | TAA |  |  |  |
| ORF18* | 14401 | 16164 | 587 | +1 | ATG | TAG | Major tail sheath protein (*Staphylococcus* phage K) | BLASTp | 0 |
| ORF19* | 16237 | 16665 | 142 | +1 | GTG | TAA | Tail protein (*Listeria* phage A511) | BLASTp | 3.00E-51 |
| ORF20 | 16762 | 16902 | 46 | +1 | ATG | TAA |  |  |  |
| ORF21 | 16945 | 17403 | 152 | +1 | ATG | TAA |  |  |  |
| ORF22 | 17416 | 17610 | 64 | +1 | ATG | TAG |  |  |  |
| ORF23 | 17692 | 18003 | 103 | +1 | ATG | TAA |  |  |  |
| ORF24 | 18135 | 18593 | 152 | +3 | ATG | TAA |  |  |  |
| ORF25 | 18637 | 19173 | 178 | +1 | ATG | TAA |  |  |  |
| ORF26* | 19226 | 23284 | 1352 | +2 | ATG | TAG | Tail lysin (*Enterococcus* phage phiEF24C) | BLASTp | 4.00E-67 |
| ORF27 | 23363 | 25789 | 808 | +2 | ATG | TAA | Tail lysin (*Enterococcus* phage phiEF24C) | BLASTp | 8.00E-101 |
| ORF28* | 25803 | 26690 | 295 | +3 | ATG | TAA | Tail lysin (*Enterococcus* phage phiEF24C) | BLASTp | 5.00E-05 |
| ORF29 | 26690 | 29236 | 848 | +2 | ATG | TAA | Glycerophosphoryl diester phosphodiesterase (*Staphylococcus* phage K) | BLASTp | 0 |
| ORF30* | 29343 | 30134 | 263 | +3 | ATG | TAA |  |  |  |
| ORF31* | 30134 | 30658 | 174 | +2 | ATG | TAA |  |  |  |
| ORF32* | 30658 | 31362 | 234 | +1 | ATG | TAG | Baseplate protein (*Staphylococcus* phage K) | BLASTp | 8.00E-135 |
| ORF33* | 31377 | 32423 | 348 | +3 | ATG | TAA | Tail protein (*Listeria* phage A511) | BLASTp | 3.00E-110 |
| ORF34 | 32444 | 35503 | 1019 | +2 | GTG | TAA | Structural protein (*Listeria* phage A511) | BLASTp | 1.00E-93 |
| ORF35* | 35614 | 36135 | 173 | +1 | ATG | TAA | Structural protein (*Listeria* phage A511) | BLASTp | 1.00E-53 |
| ORF36* | 36156 | 39614 | 1152 | +3 | ATG | TAA | Tail protein (*Listeria* phage A511) | BLASTp | 0 |
| ORF37 | 39663 | 39821 | 52 | +3 | ATG | TAG |  |  |  |
| ORF38* | 39822 | 41744 | 640 | +3 | ATG | TAA |  |  |  |
| ORF39 | 41767 | 42141 | 124 | +1 | ATG | TAA |  |  |  |
| ORF40* | 42148 | 43524 | 458 | +1 | ATG | TAG |  |  |  |
| ORF41 | 43616 | 45364 | 582 | +2 | ATG | TAG | Helicase (*Staphylococcus* phage K) | BLASTp | 0 |
| ORF42 | 45376 | 46989 | 537 | +1 | ATG | TAA | Rep protein (*Staphylococcus* phage K) | BLASTp | 0 |
| ORF43 | 46982 | 48424 | 480 | +1 | ATG | TAA | Helicase (*Staphylococcus* phage K) | BLASTp | 0 |
| ORF44 | 48503 | 49540 | 345 | +2 | ATG | TAA | Exonuclease (*Staphylococcus* phage K) | BLASTp | 0 |
| ORF45 | 49540 | 49917 | 125 | +1 | ATG | TAA |  |  |  |
| ORF46 | 49917 | 51836 | 639 | +3 | ATG | TAA | Exonuclease (*Staphylococcus* phage K) | BLASTp | 0 |
| ORF47 | 51836 | 52432 | 198 | +2 | ATG | TAG |  |  |  |
| ORF48 | 52447 | 53514 | 355 | +1 | ATG | TAG | Primase (*Staphylococcus* phage K) | BLASTp | 0 |
| ORF49 | 53581 | 53919 | 112 | +1 | ATG | TAA |  |  |  |
| ORF50 | 53919 | 54371 | 150 | +3 | ATG | TAA |  |  |  |
| ORF51 | 54358 | 54966 | 202 | +1 | ATG | TAA | Resolvase (*Enterococcus* phage phiEF24C) | BLASTp | 8.00E-44 |
| ORF52 | 54983 | 55375 | 130 | +2 | ATG | TAA | NrdI protein (*Staphylococcus* phage K) | BLASTp | 2.00E-68 |
| ORF53 | 55390 | 57504 | 704 | +1 | ATG | TAG | Ribonucleotide reductase large subunit (*Staphylococcus* phage K) | BLASTp | 0 |
| ORF54 | 57518 | 58567 | 349 | +2 | ATG | TAA | Ribonucleotide reductase minor subunit (*Staphylococcus* phage K) | BLASTp | 0 |
| ORF55 | 58585 | 58914 | 109 | +1 | ATG | TAG |  |  |  |
| ORF56 | 58898 | 59218 | 106 | +2 | ATG | TAA | Thioredoxin-like protein (*Staphylococcus* phage K) | BLASTp | 1.00E-53 |
| ORF57 | 59425 | 60021 | 198 | +1 | ATG | TAA |  |  |  |
| ORF58 | 60031 | 60336 | 101 | +1 | ATG | TAA | Integration host factor (*Staphylococcus* phage K) | BLASTp | 3.00E-51 |
| ORF59 | 60412 | 61284 | 290 | +1 | ATG | TGA | DNA polymerase (*Staphylococcus* phage K) | BLASTp | 3.00E-167 |
| ORF60 | 61450 | 61962 | 170 | +1 | GTG | TAA | Restriction endonuclease HPY99I | HHpred | 7.00E-03 |
| ORF61 | 62098 | 63441 | 447 | +1 | ATG | TAA | DNA polymerase (*Staphylococcus* phage K) | BLASTp | 0 |
| ORF62 | 63607 | 64416 | 269 | +1 | ATG | TAA | Endonuclease (*Staphylococcus* phage K) | BLASTp | 7.00E-154 |
| ORF63 | 64650 | 65510 | 286 | +3 | ATG | TAA | DNA polymerase (*Staphylococcus* phage K) | BLASTp | 1.00E-167 |
| ORF64 | 65579 | 65821 | 80 | +2 | GTG | TAA |  |  |  |
| ORF65 | 65838 | 66320 | 160 | +3 | ATG | TAA |  |  |  |
| ORF66 | 66407 | 67678 | 423 | +2 | ATG | TAA |  |  |  |
| ORF67 | 67738 | 68994 | 418 | +1 | ATG | TAA | DNA repair protein (*Staphylococcus* phage K) | BLASTp | 0 |
| ORF68 | 68998 | 69351 | 117 | +1 | ATG | TAA |  |  |  |
| ORF69 | 69338 | 70000 | 220 | +2 | ATG | TAG | Sigma factor (*Staphylococcus* phage K) | BLASTp | 1.00E-122 |
| ORF70* | 70128 | 70760 | 210 | +3 | ATG | TAA | Structural protein (*Enterococcus* phage phiEF24C) | BLASTp | 2.00E-18 |
| ORF71* | 70783 | 71295 | 170 | +1 | TTG | TAG | Major tail protein (*Staphylococcus* phage K) | BLASTp | 6.00E-89 |
| ORF72* | 71310 | 71537 | 75 | +3 | TTG | TAA |  |  |  |
| ORF73 | 71633 | 71893 | 86 | +2 | ATG | TAG |  |  |  |
| ORF74 | 71897 | 72652 | 251 | +2 | ATG | TAA |  |  |  |
| ORF75 | 72645 | 73895 | 416 | +3 | ATG | TAA | DNA repair exonuclease (*Listeria* phage A511) | BLASTp | 8.00E-78 |
| ORF76 | 73909 | 74277 | 122 | +1 | ATG | TGA |  |  |  |
| ORF77 | 74264 | 74575 | 103 | +2 | ATG | TAG |  |  |  |
| ORF78 | 74639 | 75175 | 178 | +2 | ATG | TAA |  |  |  |
| ORF79 | 75168 | 75935 | 255 | +2 | ATG | TAG |  |  |  |
| ORF80 | 75913 | 76359 | 148 | +1 | ATG | TAA |  |  |  |
| ORF81 | 76359 | 77222 | 287 | +3 | ATG | TAG |  |  |  |
| ORF82 | 77594 | 78325 | 243 | +2 | ATG | TAG |  |  |  |
| ORF83* | 78343 | 78801 | 152 | +1 | ATG | TAG |  |  |  |
| ORF84 | 78866 | 79309 | 147 | +2 | ATG | TAA |  |  |  |
| ORF85 | 79326 | 80030 | 234 | +3 | ATG | TAA |  |  |  |
| ORF86 | 80092 | 80490 | 132 | +1 | ATG | TAA |  |  |  |
| ORF87 | 80507 | 80569 | 20 | +2 | ATG | TGA |  |  |  |
| ORF88 | 80637 | 80879 | 80 | +3 | ATG | TAG |  |  |  |
| ORF89 | 80884 | 81048 | 54 | +1 | ATG | TGA |  |  |  |
| ORF90 | 81250 | 81426 | 58 | +1 | ATG | TAA |  |  |  |
| ORF91 | 81416 | 81949 | 177 | +2 | ATG | TAA |  |  |  |
| ORF92 | 81964 | 82212 | 82 | +1 | ATG | TAA |  |  |  |
| ORF93 | 82224 | 82400 | 58 | +3 | ATG | TAA |  |  |  |
| ORF94 | 82393 | 82689 | 98 | +1 | GTG | TAA |  |  |  |
| ORF95 | 82728 | 82919 | 63 | +3 | ATG | TAG |  |  |  |
| ORF96 | 82932 | 83300 | 122 | +3 | ATG | TAA |  |  |  |
| ORF97 | 83313 | 83660 | 115 | +3 | AT | TAA |  |  |  |
| ORF98 | 83660 | 83938 | 92 | +2 | ATG | TAA |  |  |  |
| ORF99 | 84008 | 84313 | 101 | +2 | ATG | TAG |  |  |  |
| ORF100 | 84328 | 84678 | 116 | +2 | ATG | TAA |  |  |  |
| ORF101 | 84678 | 85280 | 200 | +3 | ATG | TAA |  |  |  |
| ORF102 | 85294 | 85473 | 59 | +1 | ATG | TAA |  |  |  |
| ORF103 | 85477 | 85545 | 22 | +1 | ATG | TAA |  |  |  |
| ORF104 | 85609 | 85683 | 24 | +1 | ATG | TAG |  |  |  |
| ORF105 | 85700 | 86101 | 133 | +2 | ATG | TAA |  |  |  |
| ORF106 | 86103 | 86363 | 86 | +3 | ATG | TGA |  |  |  |
| ORF106.1 | 86294 | 86398 | 34 | +2 | ATG | TAA |  |  |  |
| ORF107 | 86415 | 86702 | 95 | +3 | ATG | TAG |  |  |  |
| ORF108 | 86713 | 86829 | 38 | +1 | ATG | TAG |  |  |  |
| ORF109 | 86819 | 87082 | 87 | +2 | ATG | TAA |  |  |  |
| ORF110 | 87159 | 87338 | 59 | +3 | ATG | TAA |  |  |  |
| ORF111 | 87353 | 87616 | 87 | +2 | ATG | TAA |  |  |  |
| ORF112 | 87619 | 87936 | 105 | +1 | ATG | TAA |  |  |  |
| ORF113 | 87937 | 88617 | 226 | +1 | GTG | TAA |  |  |  |
| ORF114 | 88706 | 88864 | 52 | +2 | ATG | TAA |  |  |  |
| ORF115 | 88899 | 89099 | 66 | +3 | ATG | TAA |  |  |  |
| ORF116 | 89100 | 89390 | 96 | +3 | ATG | TAA |  |  |  |
| ORF117 | 89482 | 89790 | 102 | +1 | ATG | TGA |  |  |  |
| ORF118 | 89787 | 90695 | 302 | +3 | ATG | TAA | Ribose-phosphate pyrophosphokinase (*Staphylococcus* phage K) | BLASTp | 6.00E-175 |
| ORF119 | 90713 | 92182 | 489 | +2 | ATG | TAA | Nicotinamide phosphoribosyl transferase (*Staphylococcus* phage K) | BLASTp | 0 |
| ORF120 | 92261 | 92506 | 81 | +2 | ATG | TAA |  |  |  |
| ORF121 | 92526 | 92918 | 130 | +3 | ATG | TAG |  |  |  |
| ORF122 | 92920 | 93141 | 73 | +1 | ATG | TAA |  |  |  |
| ORF123 | 93207 | 93518 | 103 | +3 | ATG | TAA |  |  |  |
| ORF124 | 93521 | 94030 | 169 | +2 | ATG | TAA | Endolysin (*Lactococcus* phage 1358) | BLASTp | 7.00E-04 |
| ORF125 | 94032 | 94361 | 109 | +3 | ATG | TAA |  |  |  |
| ORF126 | 94367 | 94561 | 64 | +2 | ATG | TAA |  |  |  |
| ORF127 | 94585 | 94899 | 104 | +1 | ATG | TAA |  |  |  |
| ORF128 | 94914 | 95081 | 55 | +3 | ATG | TAA |  |  |  |
| ORF129 | 95118 | 95219 | 33 | +3 | ATG | TAA |  |  |  |
| ORF130 | 96046 | 96345 | 99 | +1 | ATG | TAA |  |  |  |
| ORF131 | 96361 | 96546 | 61 | +1 | ATG | TAG |  |  |  |
| ORF132 | 96653 | 96943 | 96 | +2 | ATG | TAA |  |  |  |
| ORF133 | 96943 | 97230 | 95 | +1 | ATG | TAA |  |  |  |
| ORF134 | 97230 | 97523 | 97 | +3 | ATG | TAA |  |  |  |
| ORF135 | 97527 | 97784 | 85 | +3 | ATG | TAG |  |  |  |
| ORF136 | 97862 | 98101 | 79 | +2 | ATG | TAG |  |  |  |
| ORF137 | 98112 | 98459 | 115 | +3 | ATG | TGA |  |  |  |
| ORF138 | 99006 | 98668 | 112 | -3 | ATG | TAA |  |  |  |
| ORF139 | 99317 | 99625 | 102 | +2 | ATG | TAA |  |  |  |
| ORF140 | 99831 | 100115 | 94 | +3 | ATG | TAA |  |  |  |
| ORF141 | 100190 | 100381 | 63 | +1 | ATG | TAA |  |  |  |
| ORF142 | 100590 | 100456 | 44 | -1 | GTG | TAA |  |  |  |
| ORF143 | 101186 | 100698 | 162 | -1 | ATG | TAA | HNH endonuclease (*Lactococcus* phage CB13) | BLASTp | 2.00E-68 |
| ORF144 | 101354 | 101512 | 52 | +2 | ATG | TAA |  |  |  |
| ORF145 | 101582 | 101713 | 43 | +2 | ATG | TAA |  |  |  |
| ORF146 | 101881 | 102204 | 107 | +1 | ATG | TAA |  |  |  |
| ORF147 | 102304 | 102540 | 78 | +1 | ATG | TAG |  |  |  |
| ORF148 | 102620 | 103090 | 156 | +2 | ATG | TAG |  |  |  |
| ORF149 | 103149 | 103322 | 57 | +3 | ATG | TAA |  |  |  |
| ORF150 | 103322 | 103591 | 89 | +2 | ATG | TAA |  |  |  |
| ORF151 | 103667 | 103855 | 62 | +2 | ATG | TAA |  |  |  |
| ORF152 | 104425 | 104189 | 78 | -2 | ATG | TAA |  |  |  |
| ORF153 | 104912 | 104427 | 161 | -1 | ATG | TAA |  |  |  |
| ORF154 | 105332 | 104925 | 135 | -1 | ATG | TAA |  |  |  |
| ORF155 | 105763 | 105332 | 143 | -2 | ATG | TAA |  |  |  |
| ORF156 | 105957 | 105766 | 63 | -3 | ATG | TAA |  |  |  |
| ORF157 | 106439 | 105954 | 161 | -1 | ATG | TAG |  |  |  |
| ORF158 | 106863 | 106432 | 143 | -3 | ATG | TAA |  |  |  |
| ORF159 | 107419 | 106877 | 180 | -2 | ATG | TAA |  |  |  |
| ORF160 | 107919 | 107431 | 162 | -3 | ATG | TAG |  |  |  |
| ORF161 | 108330 | 107932 | 132 | -3 | ATG | TAA |  |  |  |
| ORF162 | 109034 | 108327 | 235 | -1 | ATG | TGA | Protein phosphatase (*Staphylococcus* phage G1) | BLASTp | 1.00E-134 |
| ORF163 | 109688 | 109134 | 184 | -1 | ATG | TAA |  |  |  |
| ORF164 | 110021 | 109704 | 105 | -1 | GTG | TAA |  |  |  |
| ORF165 | 111555 | 111007 | 182 | -3 | ATG | TGA |  |  |  |
| ORF166 | 111777 | 111559 | 72 | -3 | ATG | TAA |  |  |  |
| ORF167 | 111972 | 111778 | 64 | -3 | ATG | TAA |  |  |  |
| ORF168 | 112699 | 111962 | 245 | -2 | ATG | TAA |  |  |  |
| ORF169 | 112866 | 112762 | 34 | -3 | ATG | TAA |  |  |  |
| ORF170 | 113126 | 112878 | 82 | -1 | ATG | TAA |  |  |  |
| ORF171 | 113508 | 113119 | 129 | -3 | ATG | TAA |  |  |  |
| ORF172 | 113780 | 113607 | 57 | -1 | ATG | TAA |  |  |  |
| ORF173 | 114303 | 113821 | 160 | -3 | ATG | TAG |  |  |  |
| ORF174 | 114895 | 114353 | 180 | -2 | ATG | TAA |  |  |  |
| ORF175 | 115428 | 114895 | 177 | -3 | ATG | TAA |  |  |  |
| ORF176 | 115595 | 115431 | 54 | -1 | ATG | TAA |  |  |  |
| ORF177 | 115873 | 115598 | 91 | -2 | ATG | TAA |  |  |  |
| ORF178 | 116718 | 115873 | 281 | -3 | ATG | TAA |  |  |  |
| ORF179 | 117848 | 116730 | 372 | -1 | ATG | TAG | ATPase-like protein (*Staphylococcus* phage G1) | BLASTp | 0 |
| ORF180 | 118328 | 118002 | 108 | -1 | GTG | TAA |  |  |  |
| ORF181 | 118737 | 118321 | 138 | -3 | ATG | TAA |  |  |  |
| ORF182 | 119173 | 118871 | 100 | -2 | ATG | TAA | Recombinase (*Enterococcus* phage phiFL2B) | BLASTp | 9.00E-24 |
| ORF183 | 119361 | 119173 | 62 | -3 | ATG | TAA |  |  |  |
| ORF184 | 119566 | 119405 | 53 | -2 | ATG | TAA |  |  |  |
| ORF185 | 121614 | 119566 | 682 | -3 | ATG | TAA |  |  |  |
| ORF186* | 121955 | 121692 | 87 | -1 | ATG | TAA |  |  |  |
| ORF187 | 122730 | 122152 | 192 | -1 | ATG | TAG |  |  |  |
| ORF188 | 123349 | 122723 | 208 | -2 | ATG | TAA |  |  |  |
| ORF189 | 124238 | 123342 | 298 | -1 | ATG | TAA | DNA ligase (*Staphylococcus* phage K) | BLASTp | 5.00E-170 |
| ORF190 | 125271 | 124531 | 246 | -3 | ATG | TAA | PhoH-related protein (*Staphylococcus* phage K) | BLASTp | 1.00E-144 |
| ORF191 | 125937 | 125323 | 204 | -3 | ATG | TAG |  |  |  |
| ORF192 | 126378 | 125953 | 141 | -3 | ATG | TAA | Ribonuclease (*Staphylococcus* phage K) | BLASTp | 9.00E-74 |
| ORF193 | 126559 | 126368 | 63 | -2 | ATG | TAG |  |  |  |
| ORF194 | 127223 | 126582 | 213 | -1 | ATG | TAA |  |  |  |
| ORF195 | 127443 | 127213 | 76 | -3 | ATG | TAA | Transcriptional regulator (*Enterococcus* phage phiEF24C) | BLASTp | 8.00E-10 |
| ORF196 | 127673 | 127446 | 75 | -1 | ATG | TAA |  |  |  |
| ORF197 | 128475 | 127783 | 230 | -1 | ATG | TAA |  |  |  |
| ORF198 | 129297 | 128662 | 211 | -1 | ATG | TAA | HNH endonuclease | HHpred | 7.10E-18 |
| ORF199 | 130155 | 129364 | 263 | -1 | ATG | TAA |  |  |  |
| ORF200 | 130463 | 130155 | 102 | -2 | ATG | TAA |  |  |  |
| ORF201 | 131205 | 130576 | 209 | -1 | ATG | TAG | Endolysin (*Staphylococcus* phage K) | BLASTp | 1.00E-122 |
| ORF202 | 131976 | 131476 | 166 | -1 | ATG | TAA | Endonuclease (*Staphylococcus* phage K) | BLASTp | 4.00E-91 |
| ORF203 | 132939 | 132136 | 267 | -1 | ATG | TAA | Endolysin (*Staphylococcus* phage K) | BLASTp | 4.00E-155 |
| ORF204 | 133442 | 132939 | 167 | -2 | ATG | TAA | Holin (*Staphylococcus* phage K) | BLASTp | 7.00E-91 |
| ORF205 | 133712 | 133527 | 61 | -2 | ATG | TAA |  |  |  |
| ORF206 | 135477 | 135259 | 72 | -1 | ATG | TAA |  |  |  |
| ORF207 | 136164 | 135955 | 69 | -1 | ATG | TAA |  |  |  |
| ORF208 | 136509 | 136177 | 110 | -1 | TTG | TAG |  |  |  |
| ORF209 | 136848 | 136522 | 108 | -1 | TTG | TAG |  |  |  |
| ORF210 | 137147 | 136881 | 88 | -2 | ATG | TAA |  |  |  |
| ORF211 | 137288 | 137674 | 128 | +2 | TTG | TAA |  |  |  |
| ORF212 | 137652 | 137930 | 92 | +3 | ATG | TGA |  |  |  |
| ORF213 | 137927 | 138337 | 136 | +2 | TTG | TAA |  |  |  |

*Confirmed structural protein
