# Supplementary material for: Microbiological and Molecular Assessment of Bacteriophage ISP for the Control of Staphylococcus aureus
Source: PLoS One. 2011 Sep 9;6(9):e24418. doi: 10.1371/journal.pone.0024418 (PMC3170307; doi:10.1371/journal.pone.0024418)
Supplement: Table S4 — Predicted host-specific promotors of phage ISP. For each promotor the strand, the start and stop position in the genome, the −35 box, the spacer region, the −10 box and the length of the spacer region are given. (DOCX) [file pone.0024418.s008.docx]

| **Promoter** | **Strand** | **Start** | **Stop** | **-35 box** | **Spacer** | **-10 box** | **Length spacer** |
| --- | --- | --- | --- | --- | --- | --- | --- |
| P1 | + | 4530 | 4568 | TTGCTA | TATTAAATAAGAGCTAA | ATATAA | 17 |
| P2 | + | 7086 | 7114 | TTGATA | AATGTAATAACTATGAT | ATACTA | 17 |
| P3 | + | 7122 | 7150 | TTGATA | TTAATACATAAAAAATA | TTAATA | 17 |
| P4 | + | 7170 | 7198 | TTGTTA | TATTATTAACGTAAAAG | TAAATA | 17 |
| P5 | + | 16701 | 16729 | TTGACA | CTTTAAAATTTATATGT | TATTAT | 17 |
| P6 | + | 17630 | 17653 | TTGACA | AATTAAAACTAATAAAT | TATAAT | 17 |
| P7 | + | 18078 | 18106 | TTGAAT | CGGATAAGCGTAGGCTT | TATTAA | 17 |
| P8 | + | 23295 | 23323 | TTGACA | CAAGAGTAGTATCATAA | TATACT | 17 |
| P9 | + | 29271 | 29300 | TTGTAA | TTTAAACTAGTTCGTGA | TATATT | 17 |
| P10 | + | 35512 | 35540 | TTGACA | GAAAGTTAATAATATGG | TATACT | 17 |
| P11 | + | 43556 | 43584 | TTGACT | TGAAAAGGATTCTGTGG | TATACT | 17 |
| P12 | + | 48443 | 48471 | TTGACA | TTTTATATGTTAGGTGG | TATAAT | 17 |
| P13 | + | 53517 | 53545 | TTGACC | TTAGAGAAGTTTTATGT | TATACT | 17 |
| P14 | + | 59220 | 59248 | TTGGAA | TTTCCTACTATCTGTGC | TATACT | 17 |
| P15 | + | 60353 | 60381 | TTGACA | AGGTTTAAAATATATGG | TATAGT | 17 |
| P16 | + | 64492 | 64520 | TTGTAA | ACTAAAAAGCTTACAAA | TTATAG | 17 |
| P17 | + | 65519 | 65547 | TTGACA | ATATAGTTAACTTATGT | TATACT | 17 |
| P18 | + | 66341 | 66370 | TTGACA | AATATAAAAAACTATGT | TATAAT | 17 |
| P19 | + | 71573 | 71601 | TTGACA | ATTTATAATATCTATGA | TACACT | 17 |
| P20 | + | 74590 | 74618 | TTGACT | CTTTTTACTATATATGG | TATATT | 17 |
| P21 | + | 77382 | 77410 | TTGTAA | ATAGTTAAAAAATATAT | TAAAAT | 17 |
| P22 | + | 78804 | 78832 | TTGACA | GCTCCTATAGTTTATGA | TATAGT | 17 |
| P23 | + | 80583 | 80611 | TTGACT | CTCTTTTTGTTTTATGG | TATATT | 17 |
| P24 | + | 81198 | 81226 | TTGACA | AGAACAAATAAGTGTAG | TATAGT | 17 |
| P25 | + | 83948 | 83976 | TTGACA | CCTTTGTACTTTTGTAT | TATACT | 17 |
| P26 | + | 85549 | 85577 | TTGACA | ATTGAGTATACATAGGT | TATACT | 17 |
| P27 | + | 87101 | 87130 | TTGACA | TTAGGTTTCTTTTATTA | TATACT | 17 |
| P28 | + | 88648 | 88676 | TTGACA | GCAGGTATTTTTTATAG | TATACT | 17 |
| P29 | + | 92202 | 92230 | TTGACA | AAGGGAGTTTTTTATTA | TATAGT | 17 |
| P30 | + | 93157 | 93185 | TTGACT | TAGGTAGGTATCTATTA | TATAAT | 17 |
| P31 | + | 95985 | 96014 | TTGACA | ACTATGAAGCGGGTATGC | TATAAT | 18 |
| P32 | - | 99120 | 99092 | TTGACT | TCTGAATAACTATACTG | TAATAT | 17 |
| P33 | + | 99215 | 99243 | TTGACT | TTATTATCATATGGTAG | TAATAT | 17 |
| P34 | + | 99635 | 99663 | TTGCAA | TCCTCAAGCATCTATAG | TAATAT | 17 |
| P35 | + | 99725 | 99753 | TTGACA | CCTTACAAGATACATGT | TATTAT | 17 |
| P36 | + | 100490 | 100519 | TTGACA | AGGTTGTATTTTTTATGG | TATAAT | 18 |
| P37 | - | 100688 | 100660 | TTGACC | TTTTCTTTTTTCTATAG | TATACT | 17 |
| P38 | - | 101247 | 101219 | TTGACA | TTAAGACCGAATTATTA | TATAAT | 17 |
| P39 | + | 101293 | 101321 | TTGACT | TTAATATCATTATAGTT | TAATAT | 17 |
| P40 | + | 101515 | 101544 | TTGACA | ACCTAGAAACAACATGT | TAATAT | 17 |
| P41 | + | 101717 | 101745 | TTGACA | ACTTAAACAATAGATGT | TAATAT | 17 |
| P42 | + | 101813 | 101841 | TTGACA | GTCACTTGAAACCATGA | TATTAT | 17 |
| P43 | + | 102218 | 102246 | TTGACT | TTCAAGCCCTACAATGT | TATTAT | 17 |
| P44 | + | 102549 | 102577 | TTGACA | TCCTAACATATAGATGG | TAATAT | 17 |
| P45 | - | 109090 | 109062 | TTGACT | TTTTTTACTAAGTATGG | TAAGAT | 17 |
| P46 | - | 110605 | 110576 | TTGACT | ATGATTTAATATTGTATG | TGGATT | 18 |
| P47 | - | 112758 | 112730 | TTGACA | TTATTATCAATATATGT | TATTAT | 17 |
| P48 | - | 117910 | 117882 | TTGACA | AATACAAATACTTGTAA | TATAAT | 17 |
| P49 | - | 117993 | 117765 | TTGACA | ATTACCTTACCCTATGT | TAAGTT | 17 |
| P50 | - | 119494 | 119376 | TTGACA | ATAGTATCATAATATGA | TATAAT | 17 |
| P51 | - | 121693 | 121645 | TTGACA | AATATTATTTACTATGG | TATGAT | 17 |
| P52 | - | 124521 | 124493 | TTGACA | AATCCCCTTAGTTATGG | TATAAT | 17 |
| P53 | - | 127734 | 127706 | TTGACT | TCATAAGTTAACTATGC | TATAAT | 17 |
| P54 | - | 128563 | 128535 | TTGCGT | TATTTAAAGATATATGT | TATGAT | 17 |
| P55 | - | 129347 | 129319 | TTGACA | TAGGTGGTTTTTTATGC | TATAGT | 17 |
| P56 | - | 130523 | 130494 | TTGACA | AAATTAAATACATAGTGT | ATAGTT | 18 |
| P57 | - | 132025 | 131997 | TTGACA | ACATAATAACTTTCCTA | TATAGT | 17 |
| P58 | - | 135056 | 135028 | TTGACA | AGTAATAAAAATTATGT | TATAAT | 17 |
| P59 | - | 135232 | 135204 | TTGACA | AAGGTATCAGTATATGC | TATAAT | 17 |
| P60 | - | 135544 | 135516 | TTGACT | TATTTATCAATATAGTA | TATAGT | 17 |
| P61 | - | 137390 | 137362 | TTGAGT | AAATAAGGGACAAGGAA | TTAATA | 17 |
| P62 | + | 136731 | 136760 | TTGAGC | CATTTATTAATACTCTGT | CTATTC | 18 |
| P63 | + | 137075 | 137103 | TTGTAA | CACCACTAAGTATAAAA | TAATAG | 17 |
| P64 | + | 137168 | 137676 | TTGACC | TATTATTTCTAGAACTT | TTAGAT | 17 |
