# Supplementary material for: Microbiological and Molecular Assessment of Bacteriophage ISP for the Control of Staphylococcus aureus
Source: PLoS One. 2011 Sep 9;6(9):e24418. doi: 10.1371/journal.pone.0024418 (PMC3170307; doi:10.1371/journal.pone.0024418)
Supplement: Table S5 — Predicted factor-independent terminators of phage ISP. For each terminator the strand, the start and stop position in the genome, the free energy of their secundary structure and the sequence of the regulatory element (the palindromic sequence is underlined) are given. (DOCX) [file pone.0024418.s009.docx]

| **Terminator** | **Strand** | **Start** | **Stop** | **ΔG (kcal/mol)** | **Regulatory element sequence** |
| --- | --- | --- | --- | --- | --- |
| T1 | + | 4511 | 4541 | -12.00 | AAGAGGAGUAAUUACUCCUCUUUUUUUGUUU |
| T2 | + | 7038 | 7073 | -11.90 | AAGCCUAGAAUAAAUCUAGGCUUUGUUUAUUUUUUU |
| T3 | + | 10512 | 10544 | -13.30 | AGGGAUAAACUUAGGGUUUAUCCCUUUUUUAUU |
| T4 | + | 16672 | 16702 | -11.80 | AGACCAACUAAAAAGUUGGUCUUUUUUUAUU |
| T5 | + | 29240 | 29268 | -10.50 | AAAGACCUAUUAAUUUAGGUCUUUUUUU |
| T6 | + | 43523 | 43557 | -12.80 | AGACUAGGAGAAAUUUCCUAGUCUUUUUUUUUCUU |
| T7 | + | 59228 | 59276 | -23.40 | UCCUACUAUCUGUGCUAUACUAUAAUAGUACAAGGUAGUAGGAUUUUUU |
| T8 | + | 60323 | 60354 | -10.50 | AAGAAGAGAAAUAAUUCUCUUCUUUUUUUAUU |
| T9 | + | 67684 | 67710 | -9.50 | AGAGUGCCUUAGAGCACUCUUUUAUUU |
| T10 | + | 71543 | 71574 | -12.70 | AAGACCAACUAAAAAGUUGGUCUUUUUUUAUU |
| T11 | + | 74574 | 74601 | -12.50 | AGAGUCAAGUCUUUACUUGACUCUUUUU |
| T12 | + | 87091 | 87122 | -10.90 | AAGAAACCUAUUGACAUUAGGUUUCUUUUAUU |
| T13 | + | 88628 | 88666 | -16.50 | AAAUACCUGUUGACAGCCUGUUGACAGCAGGUAUUUUUU |
| T14 | + | 92192 | 92222 | -11.70 | AAACUCCCUAUUGACAAAGGGAGUUUUUUAUU |
| T15 | - | 98656 | 98620 | -15.50 | AAUACACUAGGAAUAAUAUCCUAGUGUAUUUAUUUUU |
| T16 | + | 100402 | 100440 | -13.10 | UCCCUAGGAUUAGAUUUCUAGGGAUUUUUAUUUAUUUU |
| T17 | - | 100149 | 100114 | -14.20 | GAAGCGGUUAUAUUCAACCGCUUCAUAUUUAAUUUU |
| T18 | - | 100699 | 100672 | -10.80 | AAAGAAAAGGGUUGACCUUUUCUUUUUU |
| T19 | + | 103865 | 103893 | -10.10 | AGAGGGAAUAAAAUCCCUCUUUUAUUUUU |
| T20 | - | 104185 | 104167 | -9.90 | GAGGGAUUUAAUUUCCCUCUUUUUUU |
| T21 | - | 109122 | 109092 | -11.70 | AAACACCUAUUAAUUUAAUAGGUGUUUUUUU |
| T22 | - | 110093 | 110063 | -10.20 | ACACCUAUUUAAACUAAUAGGUGUUUUUUU |
| T23 | - | 113597 | 113570 | -11.50 | AGGCUACUUUAAUUAGUAGCCUUUUUUU |
| T24 | - | 117985 | 117932 | -17.40 | UACCUUACCCUAUGUUAAGUUAUAGGUGUAAGGUAUUUUUUUUU |
| T25 | - | 118794 | 118760 | -16.90 | CACCUUGCUUGUAGCCAAGCAGGGUGUUUUUUUUU |
| T26 | - | 121704 | 121695 | -9.30 | GAAGGACUUUAAAAAGUUCUUCUUUUUUU |
| T27 | - | 127776 | 127746 | -11.70 | AAGACUAAGAUUAAUUUCUUAGUCUUUUUUU |
| T28 | - | 128671 | 128640 | -10.40 | GAGAGUGGUAAAUAUAAUUACCUCUCUUUUUUU |
| T29 | - | 129356 | 129329 | -12.60 | AACCACCUAUUGACAUAGGUGGUUUUUU |
| T30 | - | 130565 | 130535 | -12.00 | UAGACGGAUUUUAAAUCCGUCUAUUUUUUUU |
| T31 | - | 135104 | 135066 | -8.82 | AUAGAAGUAGGUAAACGUCCCUACUUCUAUAAUUUUUUU |
